# Sulphur and carbon cycling in the subduction zone mélange

**DOI:** 10.1038/s41598-018-33610-9

**Published:** 2018-10-19

**Authors:** Esther M. Schwarzenbach, Mark J. Caddick, Matthew Petroff, Benjamin C. Gill, Emily H. G. Cooperdock, Jaime D. Barnes

**Affiliations:** 1 Freie Universität Berlin, Berlin, Germany; 2 Virginia Tech, Blacksburg, USA; 3 Department of Geological Sciences, University of Texas, Austin, Texas 78712 USA; 4 Present Address: Woods Hole Oceanographic Institution, Woods Hole, MA 02543 USA

## Abstract

Subduction zones impose an important control on the geochemical cycling between the surficial and internal reservoirs of the Earth. Sulphur and carbon are transferred into Earth’s mantle by subduction of pelagic sediments and altered oceanic lithosphere. Release of oxidizing sulphate- and carbonate-bearing fluids modifies the redox state of the mantle and the chemical budget of subduction zones. Yet, the mechanisms of sulphur and carbon cycling within subduction zones are still unclear, in part because data are typically derived from arc volcanoes where fluid compositions are modified during transport through the mantle wedge. We determined the bulk rock elemental, and sulphur and carbon isotope compositions of exhumed ultramafic and metabasic rocks from Syros, Greece. Comparison of isotopic data with major and trace element compositions indicates seawater alteration and chemical exchange with sediment-derived fluids within the subduction zone channel. We show that small bodies of detached slab material are subject to metasomatic processes during exhumation, in contrast to large sequences of obducted ophiolitic sections that retain their seafloor alteration signatures. In particular, fluids circulating along the plate interface can cause sulphur mobilization during several stages of exhumation within high-pressure rocks. This takes place more pervasively in serpentinites compared to mafic rocks.

## Introduction

Subduction of altered oceanic lithosphere returns water, C, S, B, and numerous other volatiles to Earth’s mantle (e.g.^1–3^). The recycling of these species modifies the redox state of the mantle and controls the chemistry of arc volcanoes through the ascent of fluids from the slab into the sub-arc mantle^4–6^, where they ultimately control the formation of arc-related porphyry deposits^7^. However, the sources, pathways, and speciation of sulphur and carbon species during subduction are still poorly constrained. Evidence for volatile transfer from slab to mantle wedge has been primarily inferred from arc-related tracers, such as ^34^S-enriched sulphur signatures in arc volcanoes that likely originate from subducted seawater sulphate^8^, the oxidized state of arc magmas, and oxidizing, sulphate-bearing veins within porphyry deposits (see references in^9^). However, these tracers retain an imprint from chemical interaction within the sub-arc mantle and do not provide direct evidence of sulphur and carbon cycling along the slab-mantle wedge interface. Better insight into the mechanisms of fluid transfer and element cycling during subduction processes can be gained from exhumed subduction-related rocks.

During subduction, volatiles are released by sediment compaction and subsequent metamorphic reactions, with associated metamorphic mineral growth indicative of some of these reactions (e.g.^10–12^). Fluids released during metamorphic dehydration can produce complex channel networks^13,14^ and are typically thought to be focused within a narrow mélange zone along the interface of the subducting slab and the mantle wedge^15,16^. This mélange is characterized by chemically hybridized rocks and provides a window into fluid circulation within the subduction zone channel^15,17,18^. Carbon cycling within subduction zones has been the focus of numerous recent studies to constrain the long-term carbon flux between the surface and deep-Earth reservoirs (e.g.^19,20^). However, sulphur isotope data from subduction-related rocks currently consists mostly of *in situ* sulphide mineral analyses^21,22^, with bulk rock sulphur compositions of high-pressure rocks known for just a few metamorphosed serpentinite samples^23,24^. Furthermore, sulphate microphases are typically absent within both seafloor altered serpentinites and high-pressure rocks, but oxidized sulphur species are incorporated into rock-forming phyllosilicates such as serpentine^25^. Due to the difficulty in detecting this sulphate, its impact during subduction metamorphism is often neglected and thus fails to capture the behaviour of the bulk sulphur species within both seafloor altered serpentinites and high-pressure equivalents.

To better understand both sulphur and carbon fluxes within subduction complexes, we investigated exhumed mafic and ultramafic samples from Syros, Greece. Bulk rock sulphur (sulphate and sulphide) and carbon (carbonate carbon and reduced/organic carbon) species have been chemically extracted and their isotopic compositions determined. The presence of sulphur in both oxidized (sulphate) and reduced (sulphide) states in altered oceanic lithosphere makes it an ideal tool for tracking fluid sources and redox conditions, and allows us to infer mechanisms of element redistribution in the subduction channel mélange.

## Geological Setting

**Figure 1:**
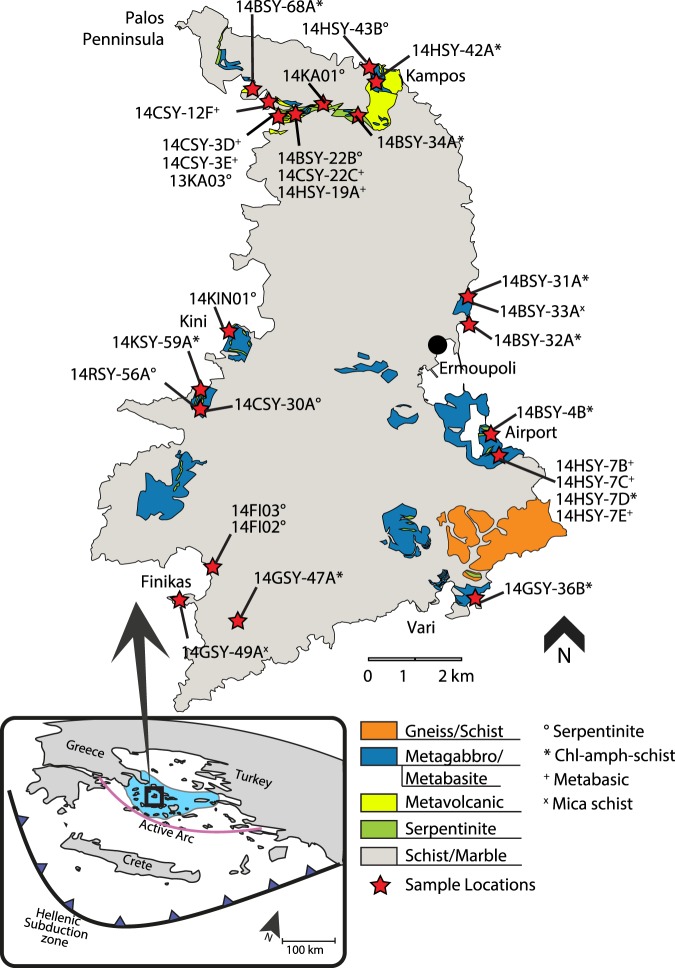
Simplified geologic map of Syros with the locations (red stars) of the studied samples. The box to the left locates the island of Syros (black box) and highlights in blue the Cycladic Blueschist Unit (CBU). Map modified after^27^.

The island of Syros is dominated by interlayered schists and marbles, interpreted as metamorphosed flysch sediments, and local blueschist- to eclogite-facies meta-ophiolitic units of metabasic to ultramafic rocks (Fig. 1) (e.g.^26,27^). These mafic and ultramafic successions represent Cretaceous oceanic lithosphere formed within the Neo-Tethys between 76–80 Ma^28,29^ that was subducted to peak P-T conditions of >15 kbar and ~500 °C^26^ by ~50–53 Ma (e.g.^10,30^). The metabasic and ultramafic lithologies occur as metre to decametre blocks of eclogite, omphacite/jadeite fels, garnet glaucophane schist, meta-gabbro and serpentinite, representing the altered components of the former oceanic lithosphere. These lithologies are surrounded by a highly sheared matrix of chlorite (±amphibole) schist and serpentinite. The metabasic and ultramafic blocks and the chlorite (±amphibole) schist matrix are thought to represent the subduction channel mélange: the deformed and metasomatized interface between the subducting slab and the overlying mantle wedge^18,31^. For this study, we investigated serpentinites, metabasic rocks and chlorite (±amphibole) schists and felses from throughout the island of Syros (Fig. 1). These represent blocks of detached lithospheric slab and their surrounding mélange matrix, allowing us to evaluate the chemical exchanges between subducted oceanic lithosphere and surrounding matrix and provide new constraints on volatile fluxes within the subduction zone channel.

## Carbon and sulphur signatures compared to altered oceanic lithosphere

**Figure 2:**
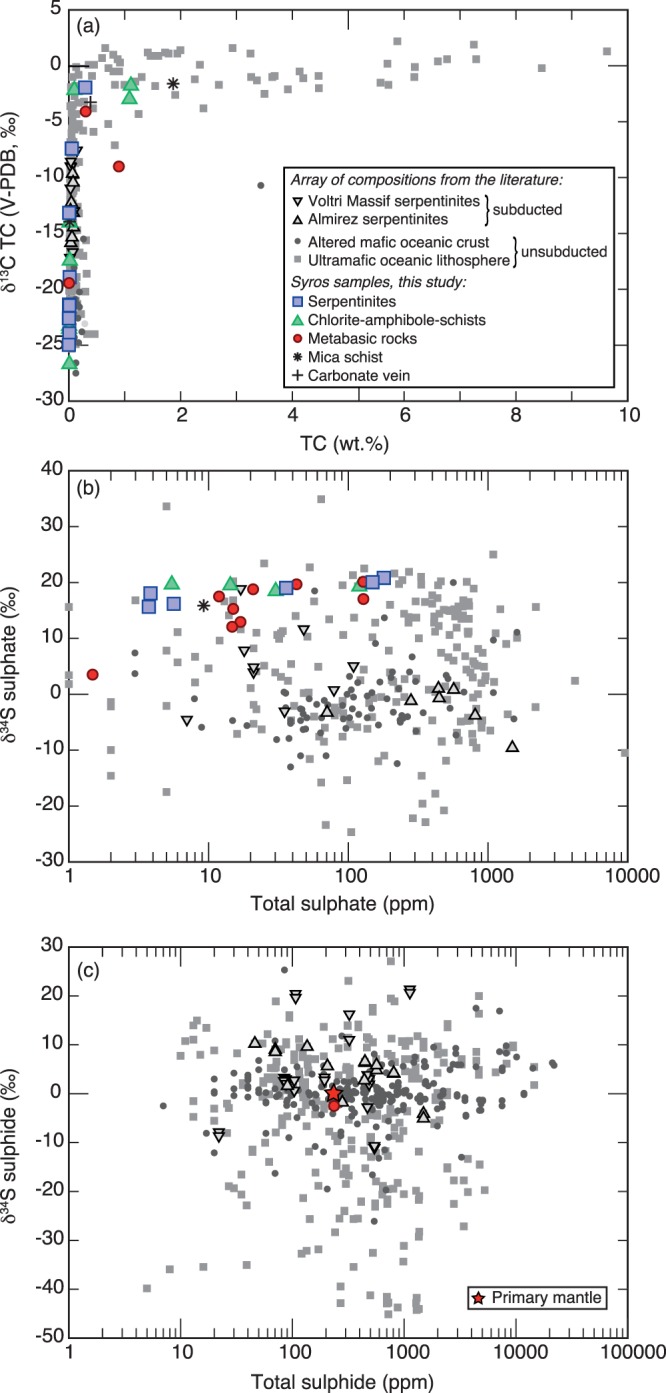
(**a**) Bulk rock carbon (total carbon, TC), (**b**) sulphate, and (**c**) sulphide isotope composition and contents of the studied samples compared to seafloor-altered serpentinites^41,54–59^, high-pressure serpentinites^23,24^, altered mafic oceanic crust^32,38,60^. Total carbon is comprised of carbonate carbon and reduced or organic carbon. Note, that sulphide contents are below detection limit in all serpentinites. Furthermore, seafloor-altered and high-pressure metamorphosed (up to eclogite facies) serpentinites from the Voltri Massif in Italy^24^ and the Cerro del Almires in Spain^23^ have ranges of carbon and sulphur signatures significantly larger than those in the Syros serpentinites and suggest preservation of the seafloor alteration signal.

**Figure 3:**
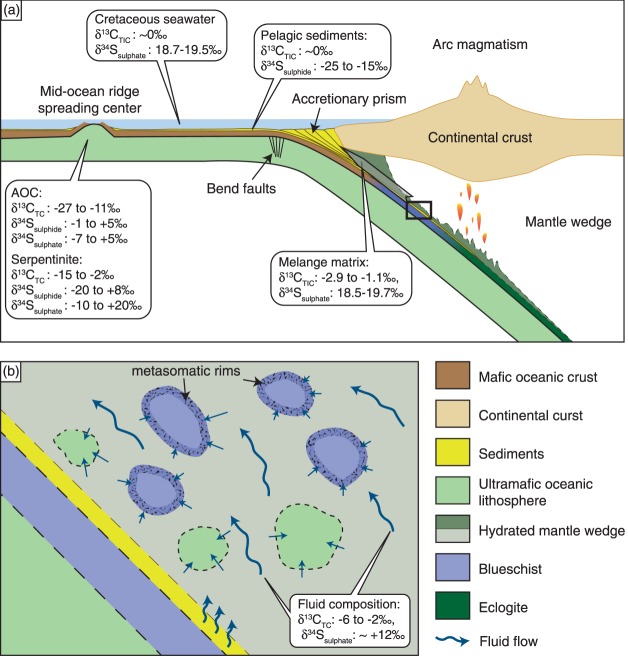
(**a**) Schematic cross-section of a subduction zone (after^15,31^) showing the average range of carbon and sulphur composition in altered oceanic crust (AOC) and serpentinites, and the isotopic composition of the dominant carbon and sulphur species in pelagic sediments, Cretaceous seawater and the mélange matrix. The grey arrow labelled ‘mélange matrix’ indicates exhumation of the metabasic and meta-ultrabasic rocks along the subduction channel and modification of the mélange matrix chemistry by mechanical mixing. (**b**) Enlargement of the processes occurring in the subduction channel (square in (**a**)) where fluids are released by dehydration of sedimentary sequences (pelagic sediments), circulate and may interact to variable degrees with detached metabasic and meta-ultrabasic blocks under blueschist-facies conditions.

Carbon and sulphur concentrations and stable isotope compositions of the investigated samples fall within the ranges measured in unmetamorphosed (unsubducted), altered oceanic lithosphere (Figs 2, 3; Tables 1, 2). However, it is striking that i) sulphide concentrations are exceptionally low (mostly below detection limit) in all of the analysed samples (only one sample contained sulphide measureable for δ^34^S_sulphide_; see Table 1) and ii) there is a positive and narrow range in δ^34^S_sulphate_ values measured in the serpentinites (15.7–20.8‰) and chlorite schists (18.5–19.7‰), whereas the metabasic samples have a larger range (δ^34^S_sulphate_ = 3.5 to 20.1‰; Fig. 2b, Table 1). These sulphur signatures are surprising since complex sulphur cycling processes during ocean floor alteration (e.g., microbial and thermochemical sulphate reduction) produce sulphur contents of up to 1 wt.% and sulphide and sulphate δ^34^S values of −45 to +27‰ (Figs 2b,c and 3)^1,32^. Previous studies suggest that subduction metamorphism does not considerably modify the sulphur isotope signatures gained during oceanic alteration, with a large range of sulphide and sulphate isotopic compositions preserved in seafloor-altered and subducted ophiolites (e.g., in high-P serpentinites in the Voltri massif, Italy; see Fig. 2)^21,22,24^. The main processes that can modify the bulk rock carbon and sulphur chemistry during subduction are metamorphic devolatilisation reactions^21,23^ or metasomatism through external fluid infiltration – with fluids produced by metamorphic dehydration reactions. In particular, dehydration of serpentinite to harzburgite and the transition from blueschist to eclogite facies have been shown to release ^34^S-enriched sulphur in the form of sulphate or SO_2_, facilitated by liberation of significant amounts of water^20,21,23^.

**Table 1:** Sulphur geochemistry of the studied samples. AVS = acid volatile sulphur (e.g., sulphide bound in pyrrhotite), CRS = chromium reducible sulphur (e.g., sulphide bound in pyrite).

Sample	Rock type	AVS (ppm)	CRS (ppm)	Sulphate S (ppm)	Total S (ppm)	SO_4_/S total	δ^34^S AVS (‰, V-CDT)	δ^34^S CRS (‰, V-CDT)	δ^34^S sulphate (‰, V-CDT)
***Serpentinites***									
14BSY-22B	serpentinite	<l.o.d.	<l.o.d.	<l.o.d.	<l.o.d.	n.d.	n.d.	n.d.	n.d.
14CSY-30A	serpentinite	<l.o.d.	<l.o.d.	<l.o.d.	<l.o.d.	n.d.	n.d.	n.d.	n.d.
14HSY-43B	serpentinite	<l.o.d.	<l.o.d.	6	6	n.d.	n.d.	n.d.	16.2
14RSY-56A	serpentinite	<l.o.d.	<l.o.d.	4	4	n.d.	n.d.	n.d.	15.7
14KA01	serpentinite	<l.o.d.	<l.o.d.	<l.o.d.	<l.o.d.	n.d.	n.d.	n.d.	n.d.
13KA03	sheared serpentinite w/talc	<l.o.d.	<l.o.d.	181	181	>99%	n.d.	n.d.	20.8
14FI02	serpentinite w/talc	<l.o.d.	<l.o.d.	36	36	>99%	n.d.	n.d.	19.0
14FI03	serpentinite w/talc	<l.o.d.	<l.o.d.	151	151	>99%	n.d.	n.d.	20.1
14KIN01	serpentinite w/talc	<l.o.d.	<l.o.d.	4	4	n.d.	n.d.	n.d.	18.1
***Metabasic samples***									
14BSY-4B	blueschist	<l.o.d.	<l.o.d.	130	130	>99%	n.d.	n.d.	17.1
14HSY-7D	blueschist	<l.o.d.	<l.o.d.	43	43	>99%	n.d.	n.d.	19.7
14BSY-31A	pillow basalt	<l.o.d.	<l.o.d.	21	21	>99%	n.d.	n.d.	18.8
14BSY-32A	metagabbro	<l.o.d.	<l.o.d.	12	12	>99%	n.d.	n.d.	17.5
14BSY-34A	basaltic dike	<l.o.d.	<l.o.d.	1	1	n.d.	n.d.	n.d.	3.5
14HSY-42A	metagabbro	5 ± 2	<l.o.d.	15	20	76	n.d.	n.d	15.3
14GSY-47A	blueschist	<l.o.d.	<l.o.d.	17	17	>99%	n.d.	n.d.	12.9
14KYY-59A	metagabbro	28 ± 5	207 ± 20	15	250	6	n.d.	−2.4	12.1
14BSY-68A	blueschist	<l.o.d.	20 ± 5	129	149	87	n.d.	n.d.	20.1
***Chlorite*** ± ***amphibole schists***									
14CSY-3D	serpentine-talc-chlorite-schist	<l.o.d.	<l.o.d.	6	6	n.d.	n.d.	n.d.	19.7
14CSY-3E	tlc-amph-chlorite-schist	<l.o.d.	<l.o.d.	<l.o.d.	<l.o.d.	n.d.	n.d.	n.d.	n.d.
14HSY-7B	chlorite schist	<l.o.d.	<l.o.d.	<l.o.d.	<l.o.d.	n.d.	n.d.	n.d.	n.d.
14HSY-7C	chlorite-amph-schist	<l.o.d.	<l.o.d.	14	14	n.d.	n.d.	n.d.	19.5
14HSY-7E	chlorite-amph-schist	<l.o.d.	<l.o.d.	121	121	>99%	n.d.	n.d.	19.3
14CSY-12F	amph-schist	<l.o.d.	<l.o.d.	30	30	>99%	n.d.	n.d.	18.5
14HSY-19A	talc-rich serpentinite/chlorite-amph-schist	<l.o.d.	<l.o.d.	<l.o.d.	<l.o.d.	n.d.	n.d.	n.d.	n.d.
14CSY-22C	chlorite-amph-schist	<l.o.d.	<l.o.d.	<l.o.d.	<l.o.d.	n.d.	n.d.	n.d.	n.d.
***Mica schists***									
14BSY-33A	mica schist	<l.o.d.	<l.o.d.	<l.o.d.	<l.o.d.	n.d.	n.d.	n.d.	n.d.
14GSY-36B	carbonate vein	<l.o.d.	<l.o.d.	83	83	>99%	n.d.	n.d.	20.8
14GSY-49A	mica schist	<l.o.d.	<l.o.d.	9	9	n.d.	n.d.	n.d.	15.9

**Table 2:** Carbon geochemistry of the studied samples.

Sample	Rock type	TC (ppm)	TIC (ppm)	TOC^a^ (ppm)	δ^13^C TC (‰, V-PDB)	δ^13^C TIC (‰, V-PDB)	δ^18^O TIC (‰, V-PDB)	δ^18^O TIC (‰, V-SMOW)	δ^13^C TOC (‰, V-PDB)	% TIC^b^
***Serpentinites***										
14BSY-22B	serpentinite	200	<l.o.d.	n.d.	−18.9	n.d.	n.d.	n.d.	−26.6	n.d.
14CSY-30A	serpentinite	136	<l.o.d.	n.d.	−24.0	n.d.	n.d.	n.d.	−26.3	n.d.
14HSY-43B	serpentinite	68	<l.o.d.	n.d.	−22.6	n.d.	n.d.	n.d.	−26.3	n.d.
14RSY-56A	serpentinite	575	482	93	−7.4	−2.2	−14.7	15.8	−26.6	84
14KA01	serpentinite	91	<l.o.d.	n.d.	−21.4	n.d.	n.d.	n.d.	−27.2	n.d.
13KA03	sheared serpentinite w/talc	155	<l.o.d.	n.d.	−21.4	n.d.	n.d.	n.d.	−24.6	n.d.
14FI02	serpentinite w/talc	2980	2297	683	−1.9	0.0	−18.7	11.7	−26.8	77
14FI03	serpentinite w/talc	117	<l.o.d.	n.d.	−13.2	n.d.	n.d.	n.d.	−27.4	n.d.
14KIN01	serpentinite w/talc	47	<l.o.d.	n.d.	−25.0	n.d.	n.d.	n.d.	−27.6	n.d.
***Metabasic samples***										
14BSY-4B	blueschist	84	<l.o.d.	n.d.	−21.8	n.d.	n.d.	n.d.	−25.8	n.d.
14HSY-7D	blueschist	<l.o.d.	<l.o.d.	n.d.	n.d.	n.d.	n.d.	n.d.	n.d.	n.d.
14BSY-31A	pillow basalt	<l.o.d.	<l.o.d.	n.d.	n.d.	n.d.	n.d.	n.d.	n.d.	n.d.
14BSY-32A	metagabbro	<l.o.d.	<l.o.d.	n.d.	n.d.	n.d.	n.d.	n.d.	n.d.	n.d.
14BSY-34A	basaltic dike	<l.o.d.	<l.o.d.	n.d.	n.d.	n.d.	n.d.	n.d.	−28.4	n.d.
14HSY-42A	metagabbro	96	<l.o.d.	n.d.	−19.4	n.d.	n.d.	n.d.	−25.8	n.d.
14GSY-47A	blueschist	3060	2945	115	−4.1	−0.7	−19.8	10.5	−24.8	96
14KYY-59A	metagabbro	<l.o.d.	<l.o.d.	n.d.	n.d.	n.d.	n.d.	n.d.	n.d.	n.d.
14BSY-68A	blueschist	9000	10044	<l.o.d.	−9.0	−7.4	−10.0	20.6	−24.5	>99
***Chlorite*** ± ***amphibole schists***										
14CSY-3D	serpentine-talc-chlorite-schist	1030	1030	<l.o.d.	−2.2	−1.1	−17.8	12.5	−26.7	>99
14CSY-3E	tlc-amph-chlorite-schist	94	<l.o.d.	n.d.	−23.3	n.d.	n.d.	n.d.	−27.4	n.d.
14HSY-7B	chlorite schist	98	<l.o.d.	n.d.	−23.5	n.d.	n.d.	n.d.	n.d.	n.d.
14HSY-7C	chlorite-amph-schist	144	<l.o.d.	n.d.	−26.7	n.d.	n.d.	n.d.	−27.8	n.d.
14HSY-7E	chlorite-amph-schist	10900	10880	20	−3.0	−2.9	−10.3	20.3	−26.4	>99
14CSY-12F	chlorite-amph-schist	11160	10600	560	−1.8	−1.4	−10.6	19.9	−26.9	95
14HSY-19A	talc-rich serpentinite/chlorite-amph-schist	178	<l.o.d.	n.d.	−14.0	n.d.	n.d.	n.d.	−26.8	n.d.
14CSY-22C	chlorite-amph-schist	223	<l.o.d.	n.d.	−17.4	n.d.	n.d.	n.d.	−26.5	n.d.
***Mica schists***										
14BSY-33A	mica schist	18720	18010	710	−1.6	−1.6	−19.3	11.1	−27.8	96
14GSY-36B	carbonate vein	3920	3739	181	−3.3	−1.0	−14.0	16.5	n.d.	95
14GSY-49A	mica schist	177	<l.o.d.	n.d.	−13.9	n.d.	n.d.	n.d.	−23.4	n.d.

TC = total carbon; TIC = total inorganic carbon; TOC = total organic carbon (includes all reduced or non-carbonate carbon). ^a^TOC = TC - TIC. ^b^%TIC = TIC/TC.

**Figure 4:**
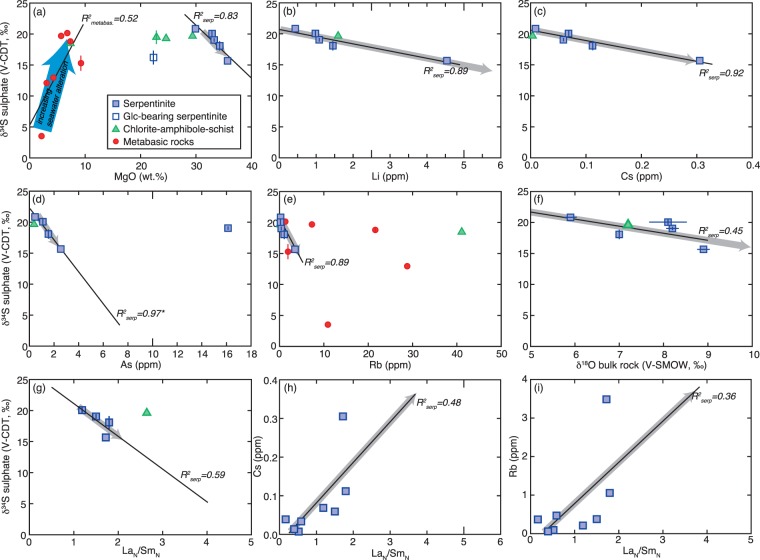
(**a**–**f**) Selected element concentrations versus δ^34^S_sulphate_ showing a distinct negative correlation, and La_N_/Sm_N_ against (**g**) δ^34^S_sulphate_ (**h**) Cs, and (**i**) Rb. All reflect mixing between a seawater-dominated fluid and a sediment-derived component with the grey arrows indicate increasing input of sedimentary-derived fluid. R^2^ (black line) is calculated for the serpentinites only. For As the R^2^-value (R^2^_serp_ = 0.97*) excludes the outlier, as R^2^ for all data would not be meaningful.

The mafic blocks from Syros are interpreted to be of oceanic origin based on bulk rock δ^18^O and δD values, retaining an imprint of seafloor alteration^33,34^. Carbon and sulphur in the metabasic blocks in this study overlap with altered oceanic crust (AOC) in terms of their isotopic composition, though sulphide contents are significantly lower (Fig. 2). Our results show positive correlations between Mg and δ^34^S_sulphate_ (Fig. 4a) and weak positive correlations between SO_4_-contents and MgO, K_2_O, Sr, and loss on ignition (bulk rock volatile content; not shown), suggesting increasing seawater alteration with increasing seawater sulphate incorporation^35^. However, some mafic blocks demonstrate interaction with secondary fluids. For example, blackwall reaction rinds at the contact between metabasic and ultramafic blocks and their surrounding mélange matrix are inferred to reflect fluid exchange during exhumation^18,31^. These rinds include tourmaline-bearing mafic rocks that formed during exhumation^31^. One tourmaline-bearing metagabbro studied here yields a δ^34^S_sulphate_ of 12.1‰, suggesting that fluids circulating during exhumation had an isotopic composition similar to this value, i.e., considerably lower than seawater sulphate compositions during the late Cretaceous (δ^34^S_seawater-sulphate_ ≈ 18.7–19.5‰^36^). Furthermore, the low sulphide contents provide strong evidence for metasomatism by an oxidizing fluid. Even though metamorphic devolatilisation reactions can cause H_2_S loss – primarily during blueschist-eclogite transition^21^, we consider it more likely that partial sulphide loss and local overprinting of the seafloor alteration signature was associated with metasomatism since these rocks did not reach eclogite-facies conditions. An even later stage of fluid circulation is recorded in two blueschist samples with distinctively higher total carbon (TC) contents of 0.3 to 0.9 wt.% (with δ^13^C of TIC [total inorganic carbon] of −0.7 and −7.4‰) – reflected by the presence of late carbonate veins – which is higher than average TC contents in AOC of ~0.21 wt.% (Fig. 2a)^37,38^. The origin and impact of these secondary fluids is discussed below.

Similarly, the serpentinites from Syros record a complex fluid history. Cooperdock *et al*.^39^ report δ^18^O and δD values of Syros serpentinites within the range of abyssal serpentinites. However, based on these data interaction with slab-derived fluids is possible. While our carbon data overlaps with abyssal serpentinites, the sulphur geochemistry of Syros serpentinites is distinctly different, with sulphide contents for all samples below detection limit (Fig. 2). Such low sulphide contents and δ^34^S_sulphate_ values close to seawater (modern δ^34^S_seawater-sulphate_ ≈ +21.15‰^40^) have previously only been observed in the active serpentinizing system of the Atlantis Massif along the Mid-Atlantic Ridge. There, carbonate and sulphate are actively incorporated into serpentinites by extensive seawater-peridotite interaction, with high water-rock ratios resulting in the oxidation of sulphide minerals^41^. In addition to abyssal metasomatism, carbonate and sulphate may be incorporated during fault-bend serpentinization by oxidizing fluids near the seawater-sediment interface along the trench prior to their subduction (Fig. 3). Recent studies have suggested that bending of the subducting plate and associated faulting facilitates fluid influx deep into the lithospheric mantle^42^. These areas may be subject to extensive serpentinization, sequestering significant quantities of water, which is eventually transferred into Earth’s interior^42^. Simultaneously, oxidizing fluids could have removed sulphide minerals formed during early phases of serpentinization near an oceanic spreading centre. Though sulphate and carbonate were possibly incorporated during mid-ocean ridge or bend fault serpentinization, considering the metasomatic rinds on the metabasic samples^18,31^ and comparing with other subducted serpentinites (e.g., Voltri Massif serpentinites; Fig. 2) it is more likely that the serpentinites were also affected by metamorphic or metasomatic reactions during subduction and/or exhumation to modify the sulphur and carbon compositions.

## Sulphur and carbon cycling in the subduction zone channel

The serpentinites studied here lack mineralogical evidence for having experienced significant dehydration. Thus, the low sulphur contents cannot primarily be ascribed to devolatilization reactions, but likely reflect external fluid ingress. Based on δ^18^O and δD values and fluid-mobile trace element compositions of serpentinites from Syros (including those studied here), a chemical exchange between ophiolitic blocks and the surrounding matrix has been proposed^39^. In addition, the sulphur geochemistry of the analysed serpentinites indicates two endmember fluid compositions that interacted with these rocks: a seawater-sulphate-like fluid and a fluid characterized by lower δ^34^S_sulphate_ values (Fig. 4). In the case of the Syros serpentinites, seawater sulphate incorporation either during serpentinization near a mid-ocean ridge spreading centre^41^ or by bend-fault serpentinization (Fig. 3a) would have occurred in the Late Cretaceous with seawater δ^34^S_sulphate_ values close to ~18.7–19.5‰^36^. The second endmember composition (with lower δ^34^S_sulphate_ values) is associated with elevated concentrations in fluid-mobile elements (FME), particularly Rb, Li, and Cs (Fig. 4), which we interpret as reflecting secondary, external fluid ingress.

Even though FMEs are typically enriched in most serpentinites, independent of geotectonic origin, abnormal enrichments of certain elements document different fluid sources^3^. Overall, FMEs such as Rb, Sr, Ba, Li, As, Cs, Pb, and U are liberated during metamorphic dehydration reactions, whereas As, Cs, and U are characteristic of a sedimentary fluid input^3,43^. At the same time, the largest reservoir of ^32^S-enriched sulphur within subduction zones is pelagic sediments that contain significant amounts of biogenic sulphide (with average δ^34^S_sulphide_ of approximately −25 to −15‰^44,45^). In particular, carbonaceous shales are considered to produce the most H_2_S-rich (and ^32^S-enriched) fluids upon metamorphism^46^. Even though sulphur is also stored within AOC, dehydration of carbonaceous shales produces one to two orders of magnitude higher concentrations of H_2_S in metamorphic fluids compared to mafic rocks^46^. Furthermore, a sedimentary fluid input is suggested in our data by negative correlations between δ^34^S_sulphate_ and both Mg and, to a lesser extent, δ^18^O (Fig. 4a,f). Sediments are enriched in MgO and have δ^18^O_clay_ values of +10 to +30‰^44^. Thus, their dehydration results in Mg-rich and ^18^O-enriched fluids shifting the oceanic signature to higher MgO contents and δ^18^O values, respectively. Additionally, we observe a trend towards high (La/Sm)_N_ at low (Ba/Th), as well as positive correlations between Cs and (La/Sm)_N_ (Fig. 4h) and Rb and (La/Sm)_N_ (Fig. 4i), which is attributable to the input of a sedimentary component within the mélange zone^47^. Thus, the studied serpentinites record numerous lines of evidence for a metasomatic overprint by a fluid produced by metamorphic dehydration of sedimentary sequences.

In contrast to the serpentinites and metabasic samples, the chlorite schists (representing the mélange matrix) have higher TC contents (up to 1.8 wt.%) with δ^13^C_TIC_ values of −2.9 to −1.1‰ (Table 2), and an exceptionally narrow range in δ^34^S_sulphate_ (18.5–19.7‰; Table 1). The sulphur compositions match seawater sulphate compositions of Late Cretaceous age (δ^34^S ≈ 18.7–19.5‰^36^) and imply a seawater-dominated component. Their rare earth element (REE) concentrations are higher than in the serpentinites (see suppl. mat.), but in most samples their FME concentrations are lower. This indicates a different origin of these rocks to the serpentinites. Chlorite schists in subduction zones have previously been described as the result of metasomatic and mechanical mixing at the contact between metabasic rocks and serpentinites^47^ and at shallow depth associated with exhumation as proposed by geodynamic modelling^48^. Mechanical mixing explains the highly variable trace element patterns of the chlorite schists (see suppl. Fig. S4-1), reflecting heterogeneous incorporation of mafic and ultramafic material. The presence of carbonate (dolomite and calcite) as pockets, layers or veins (see Suppl. Mat.) indicates that sedimentary material was also incorporated. In addition, fluids were locally focused through the mélange matrix producing carbonate veins, which are locally also preserved in the blueschists. These veins have δ^34^S_sulphate_ values of +20.1 to +20.8‰ and δ^13^C_TIC_ values of −7.4 to −1.0‰, and may have formed from fluids produced by compaction of younger sedimentary sequences that were isotopically heavier in their sulphur composition. This agrees well with exhumation ages from Syros of ~25–20 Ma^27^, at which time δ^34^S_seawater-sulphate_ was ~+21‰^36^. Thus, the late carbonate veins may be associated with late stage exhumation processes.

## Geotectonic Evolution

The serpentinite samples studied here have previously been inferred to be of abyssal origin^39^. Using oxygen and hydrogen isotopes, and major and trace element geochemistry, Cooperdock *et al*.^39^ excluded a potential origin from the fore-arc mantle wedge, as well as the influence of meteoric water. The carbon and sulphur geochemistry of the studied mafic and ultramafic samples reflects several stages of water-rock interaction by fluids with different origins: Cretaceous seawater and metamorphic dehydration. Even though the samples studied here overlap with the carbon and sulphur signatures found in seafloor altered mafic rocks, the lack of sulphide in almost all studied samples – and thus orders of magnitude lower sulphide contents compared to abyssal serpentinites – suggests sulphide removal after alteration on the seafloor, most likely during exhumation (Fig. 3). On Syros, blackwall alteration rinds and tourmaline-bearing mafic rocks that formed during exhumation record retrograde metasomatic overprinting at depths of ~25 km and temperatures ~400 °C with a fluid that was slab-derived, acidic, and unusually Li-rich^33^. The isotopic value of the tourmaline-bearing metagabbro and the negative correlation between Li contents and δ^34^S_sulphate_ (Fig. 4b) suggests that interaction of the serpentinites with external fluids most likely took place during the same stages of exhumation. Most likely, water-rock ratios were sufficiently low to allow for the preservation of the seafloor δ^18^O and δD alteration signature. Furthermore, the abundance of hematite in the blueschists (see suppl. material) and the low sulphide contents implicate an oxidizing fluid. Oxidation of sulphide to sulphate is associated with minor isotope fractionation^49^. Thus, in the metabasic samples oxidation of sulphide with δ^34^S values as low as −2.4‰ – which is within the range of sulphide found within AOC (Figs 2c, 3) – possibly generated δ^34^S_sulphate_ values of 3.5 to 20.1‰ (Table 1). In the serpentinites, these fluids would have oxidized the sulphide – if present – entirely.

In contrast, the composition of the chlorite-amphibole schists documents carbon and sulphur incorporation at shallower depth, with an isotopic signature dominated by a seawater component. The origin of these rocks suggests mechanical incorporation of trace amounts of seawater sulphate and carbonate, either from AOC, sediments, or serpentinites^47^. Even though CO_2_-rich fluids could have been focused through these rocks, the low sulphur contents indicate that these lithologies did not serve as primary fluid pathways for sulphur transfer within the subduction zone channel unless fluids were in chemical and isotopic equilibrium with the rock and thus left little record of fluid-rock interaction. However, as seawater circulation results in significant addition of seawater sulphate into abyssal serpentinites^41^ and incorporation of the sulphate into the serpentine structure^25^, we infer that sulphur concentrations in the fluid within the subduction channel were most likely very low. This also agrees with the low solubility of anhydrite at these P and T conditions, which inhibits extensive sulphur mobility^9^.

We advocate that although large sequences of serpentinite can preserve sulphur and carbon signatures of the subducted slab (e.g., in the Voltri Massif serpentinites)^24^, small bodies of decoupled oceanic lithosphere may be subject to considerable fluid infiltration during exhumation. Most likely, sulphur geochemical modification and sulphur transfer to the sub-arc mantle occur near the transition from blueschist- to eclogite-facies conditions, driven by dehydration reactions^20,21,23^. If these conditions are not reached, oxidizing fluids circulating during exhumation can induce sulphide oxidation and removal of sulphide phases. In particular, sulphide dissolution is enhanced during deformation and the input of highly saline fluids, which also increases sulphate solubility^9,46,50^. Additional sulphur transfer takes place during late stages of exhumation upon interaction with large quantities of saline, oxidizing and seawater-sulphate-dominated fluids liberated during compaction of sediments and expulsion of pore fluid during initial stages of subduction within the accretionary prism^51^. These fluids are documented by late-stage carbonate veins with δ^34^S_sulphate_ around +20.8‰ and may have affected highly sheared serpentinites, providing an explanation for δ^34^S values higher than Cretaceous seawater sulphate (see Table 1) because release of seawater of <50 Ma age would be characterized by δ^34^S_sulphate_ values between 21.5–22.5‰^36^. Oxidation by such late, seawater-dominated saline fluids could have additionally caused sulphate addition and local sulphide loss. Interestingly, metamorphic dehydration fluids seem to have more extensively affected the serpentinites than the metabasic samples, where interaction with dehydration fluids was restricted to the metasomatic rinds (Fig. 3). In contrast with these sulphur data, carbon results do not readily imply a suite of fluid sources and cannot easily be used as a tracer for specific fluid interactions. Therefore, we conclude that carbon was largely immobile during metamorphism at these P-T conditions.

The lack of sulphide in the majority of our analysed samples, which contain orders of magnitude lower sulphide concentrations than typical seafloor altered oceanic lithosphere (Fig. 3b,c), documents that sulphur can be mobilized at various stages and by several processes during evolution within a subduction zone. Furthermore, we show that oxidizing fluids play a central role in the mobilization of sulphur, particularly in smaller blocks of detached slab material, and that numerous lithologies can provide a sulphur source during subduction zone processes. This study also highlights the importance of constraining bulk rock sulphur and carbon compositions, which can reveal important aspects of chemical cycles within subduction zones and allows the tracking of bulk element fluxes between the surface and Earth’s deep reservoirs.

## Methods

### Sample preparation

For all samples thin section blocks were cut for thin sections for optical microscopy and electron microprobe (EMP) analyses. Bulk rock powders were prepared for determination of the bulk rock carbon and sulphur geochemistry. Prior to crushing the samples, the outermost 1–2 cm of the rock samples were cut away to remove contamination from weathering. The samples were cleaned in an ultrasonic bath prior to powdering them with a shatter box using an alumina dish. Sample preparation was carried out at the Department of Geosciences at Virginia Tech.

### Mineralogy

The mineralogy of the studied samples was determined by transmitted and reflected light microscopy. On selected samples EMP analyses were performed in order to identify the mineral chemistry, specifically determining the carbonate phases. EMP analyses were carried out on a JEOL superprobe electron microprobe at the Geological department at the Freie Universität Berlin using 15 kV acceleration potential, a 20 nA current and a 1 μm beam size using natural and synthetic mineral standards. Relative analytical error is better than 1% (1σ) except for element contents <1 wt.%, where the analytical error is better than 10% (1σ).

### Major and trace element compositions

Major element concentrations of lithiumtetraborate fluxed fusion glasses were analysed with a PANalytical 2404×-ray fluorescence spectrometer at the Department of Earth and Environment, Franklin Marshall College (following the procedures of^52^). Volatile contents were determined by loss on ignition. Concentrations of some trace elements were determined from pressed whole rock powders. A subset of samples has been determined by Cooperdock *et al*.^39^ (marked with * in Table S4).

### Determination of the carbon geochemistry

Carbon was detected either as oxidized/inorganic carbon (TIC), reduced/organic carbon (TOC), or as the total carbon (TC) composition of the bulk rock. For all samples TC and TIC contents, and δ^13^C of TC, δ^13^C of TIC and δ^13^C of TOC were analysed. All carbon and oxygen analyses were carried out at the Department of Geosciences at Virginia Tech. TC contents, δ^13^C_TC_ and δ^13^C_TOC_ were measured on a Vario ISOTOPE elemental analyser (EA) coupled to an Isoprime 100 isotope ratio mass spectrometer (IRMS). For analyses of δ^13^C_TOC_, bulk rock samples were reacted for three days with 3 N HCl to remove all acid soluble carbon (e.g., calcite, dolomite). They were then rinsed with H_2_O, dried at 40 °C in the oven and homogenized in the agate mortar. TC and TOC isotope values are reported in the standard δ-notation relative to the Vienna-Pee Dee Belemnite (V-PDB) standard and calibrated to this scale using international [IAEA-CH-6 (sucrose; δ^13^C = −10.449‰) and IAEA-CH-7 (polyethylene; δ^13^C = −32.151‰)] and commercial standards [elemental microanalysis wheat flour; δ^13^C = −27.21‰]. Reproducibility of δ^13^C_TC_ and δ^13^C_TOC_ is better than 0.1‰.

The δ^13^C_TIC_ and δ^18^O_TIC_ and TIC contents were analysed on a MultiFlowGeo headspace sampler attached to an Isoprime 100 IRMS. Samples were prepared in septum vials, flushed with helium and acidified with phosphoric acid. Samples were then reacted for at least 3 hours at room temperature. Carbon and oxygen isotope values are reported in the standard δ-notation relative to the V-PDB standard and calibrated to this scale using the international standards IAEA-CO-1 (marble; δ^13^C = +2.492‰, δ^18^O = −2.4‰), IAEA-CO-9 (BaCO3; δ^13^C = −47.321‰, δ^18^O = −15.6‰) and NBS18 (calcite, δ^13^C = −5.014‰, δ^18^O = −23.2‰). Reproducibility for the analysis of the samples was better than ±0.07‰ for δ^13^C and better than ±0.3‰ for δ^18^O.

### Determination of the sulphur geochemistry

Sulphur extractions were carried out by reacting 22–28 g of bulk rock powder to determine the acid volatile sulphide (AVS, typically bound within monosulphides such as pyrrhotite), the chromium reducible sulphide (CRS, typically bound within disulphides such as pyrite) and the sulphate fraction (e.g., anhydrite). Extraction of the sulphur components was carried out following a modified version of the methods of Canfield *et al*.^53^. In a first step, AVS was extracted by reacting the bulk rock powder with 6 N HCl in an inert N_2_-atmosphere and in a second step the residual sample was reacted with an acidified CrCl_2_ solution to extract the CRS fraction. In both cases the liberated H_2_S was precipitated as ZnS in a zinc acetate solution and subsequently converted to Ag_2_S through reaction with a 0.1 M AgNO_3_ solution. The sulphate fraction was recovered by reacting the solution from the AVS extraction with BaCl_2_ to form BaSO_4_. Amounts of AVS, CRS and sulphate were determined gravimetrically and corrected based on the sulphur content of the precipitate as determined on the EA, since co-precipitation of other phases during the wet chemical extraction could not be completely prevented. Detection limit for sulphate and sulphide depend on the amount of sample powder processed; for 25 g of sample powder 1 ppm sulphate (corresponding to 0.0002 g of BaSO_4_ extract) and 3 ppm sulphide (corresponding to 0.0002 g of Ag_2_S extract) are still detectable.

The isotopic composition of the AVS, CRS and sulphate were determined on a Vario ISOTOPE EA attached to an Isoprime 100 IRMS. To ensure complete combustion during EA-analyses, vanadium pentoxide (V_2_O_5_) was added to the samples. Sulphur isotope values are reported in standard δ-notation relative to the Vienna-Canyon Diablo Troilite (V-CDT) standard. During measurements the international sulphide (Ag_2_S) standards IAEA-S-1 (δ^34^S = −0.3‰), IAEA-S-2 (δ^34^S = +22.7‰) and IAEA-S-3 (δ^34^S = −32.3‰) and the sulphate (BaSO_4_) standards IAEA-SO-5 (δ^34^S = +0.5‰), IAEA-SO-6 (δ^34^S = −34.1‰), and NBS127 (δ^34^S = +20.3‰) were used to place our samples on the V-CDT scale. Reproducibility is better than 0.2‰ for all sulphur analyses (samples and standards), and the relative precision of sulphur contents is within 3% determined by multiple extractions of the same sample.

## Electronic supplementary material

Supplementary Material

## Data Availability

All data generated or analysed during this study are included in this published article (and its Supplementary Information files).
