# Supplementary material for: Sulphur and carbon cycling in the subduction zone mélange
Source: Sci Rep. 2018 Oct 19;8:15517. doi: 10.1038/s41598-018-33610-9 (PMC6195527; doi:10.1038/s41598-018-33610-9)
Supplement: Supplementary file 1 — Supplementary Material [file 41598_2018_33610_MOESM1_ESM.pdf]

Supplementary material to "*Sulphur and carbon cycling in the subduction zone mélange*"

Esther M. Schwarzenbach[1,2*], Mark J. Caddick[2], Matthew Petroff[2], Benjamin C. Gill[2], Emily H. G. Cooperdock[3+], Jaime D. Barnes[3]

1 Freie Universität Berlin, Berlin, Germany

2 Virginia Tech, Blacksburg, USA

3 Department of Geological Sciences, University of Texas, Austin, Texas 78712, USA

*Corresponding author e-mail: esther.schwarzenbach@fu-berlin.de

+ Now at Woods Hole Oceanographic Institution, Woods Hole, MA, 02543, USA

1 Sample description

Table S1 includes the sampling location and the description of the ultramafic and mafic samples as determined by optical microscopy.

Table S1. Description of the mafic and ultramafic samples.

Sample (1)	Coordinates N	Coordinates E	Location	Description	Rock type (2)
Serpentinites					
14BSY-22B*	37°29'36.2"	024°54'26.4"	Kampos	Massive, fine-grained serpentinite with bastites	serpentinite
14CSY-30A*	37°25'48.7"	024°52'56.2"	Kini	Fine-grained, talc-bearing serpentinite, locally pseudomorphs of pyx preserved	serpentinite
14HSY-43B	37°30'06.1"	024°55'34.6"	Kampos	Serpentinite overgrown by zoned glaucophane needles and hematite	serpentinite
14RSY-56A*	37°25'50.6"	024°52'48.2"	Kini	Talc-bearing, fine-grained serpentinite with carbonate as pockets	serpentinite
14KA01*	37°29'36.42"	024°54'35.21"	Kampos	Massive serpentinite with pseudomorphs	serpentinite
13KA03*	37°29'24.69"	024°54'6.13"	Kampos	Sheard serpentinite	serpentinite w/ talc
14FI02*	37°23'32.96"	024°53'5.93"	Finikas	Foliated fine-grained serpentinite	serpentinite w/ talc
14FI03*	37°23'33.00"	024°53'5.96"	Finikas	Foliated fine-grained serpentinite	serpentinite w/ talc
14KIN01*	37°26'37.68"	024°53'20.40"	Kini	Massive serpentinite with pseudomorphs	serpentinite w/ talc
Metabasic samples					
14BSY-4B	37°25'21.8"	024°57'16.4"	Airport	Blueschist	blueschist
14HSY-7D	37°25'08.7"	024°57'40.3"	Airport	Primary HP blueschist w/ omphacite	blueschist
14BSY-31A	37°27'10.1"	024°56'55.0"	Ermoupoli	Pillow basalt w/ small gt (FLOAT)	pillow basalt
14BSY-32A	37°27'02.1"	024°56'57.2"	Ermoupoli	Metagabbro w/ gt, felsic dike	metagabbro
14BSY-34A	37°29'30.4"	024°55'04.7"	Kampos	Basaltic dike w/ albite cracks through metagabbro	basaltic dike
14HSY-42A	37°30'01.4"	024°55'25.1"	Kampos	Metagabbro w/ pegmatitic pyroxenes	metagabbro
14GSY-47A	37°22'47.2"	024°53'25.1"	Finikas	Micaceous retrograded b.s.	blueschist
14KYY-59A	37°25'52.1"	024°52'54.8"	Kini	Altered metagabbro with pegmatitic tourmaline, eclogite facies (FLOAT)	metagabbro
14BSY-68A	37°29'19.0"	024°54'03.5"	Kampos	Blueschist w/ cm lawsonite, carbonate vein, pinhead gt	blueschist
Chlorite ± amphibole schists					
14CSY-3D*	37°29'34.1"	024°54'05.1"	Kampos	Deformed serp-talc-chl-schist with euhedral hematite overgrowing the groundmass	serpentine-talc-chlorite-schist
14CSY-3E*	37°29'34.1"	024°54'05.1"	Kampos	Schist with layers of serp, chl, amph-dominated areas, opaque phases overgrowing the groundmass	tlc-amph-chlorite-schist
14HSY-7B*	37°25'08.7"	024°57'40.3"	Airport	Fine-grained chlorite-schist with layers of carbonate	Chlorite schist
14HSY-7C	37°25'08.7"	024°57'40.3"	Airport	Chlorite-amph-schist with amphibole overgrowing chlorite	Chlorite-amph-schist
14HSY-7E	37°25'08.7"	024°57'40.3"	Airport	Dolomite and serpentine-bearing chlorite-amphibole schist.	Chlorite-amph-schist
14CSY-12F	37°29'43.5"	024°53'34.7"	Kampos	Fine-grained amph-schist with locally large, euhedral hematite	Chlorite-amph-schist
14HSY-19A*	37°29'36.1"	024°54'29.6"	Kampos	Deformed, talc-rich serpentinite interlayered with chlorite-amph-rich layers	talc-rich serpentinite / chlorite-amph-schist
14CSY-22C	37°29'36.2"	024°54'26.4"	Kampos	Very fine-grained chlorite-amph-schist, folded, opaque phases overgrowing the groundmass and locally amph needles are overgrowing eralier formed, fine-grained amphibole	Chlorite-amph-schist
Mica schists					
14BSY-33A	37°27'04.4"	024°56'56.9"	Ermoupoli	Mica schist with euhedral dolomite	mica schist
14GSY-36B	37°23'11.9"	024°57'03.1"	Vari	Late stage carbonate vein	carbonate vein
14GSY-49A	37°23'12.1"	024°52'36.4"	Finikas	Mica schist w/ gt, glaucophane	mica schist

*(1) * denotes samples analyzed for $\delta^{18}O_{WR}$ and δD_{WR} presented in Cooperdock et al., 2018.*

(2) Distinction between blueschist, metagabbro and pillow basalt is based on textural differences.

2 Hand sample images and thin section photographs

Figures 2-1 to 2-12 show hand sample images and thin section photographs of selected samples investigated for this study.

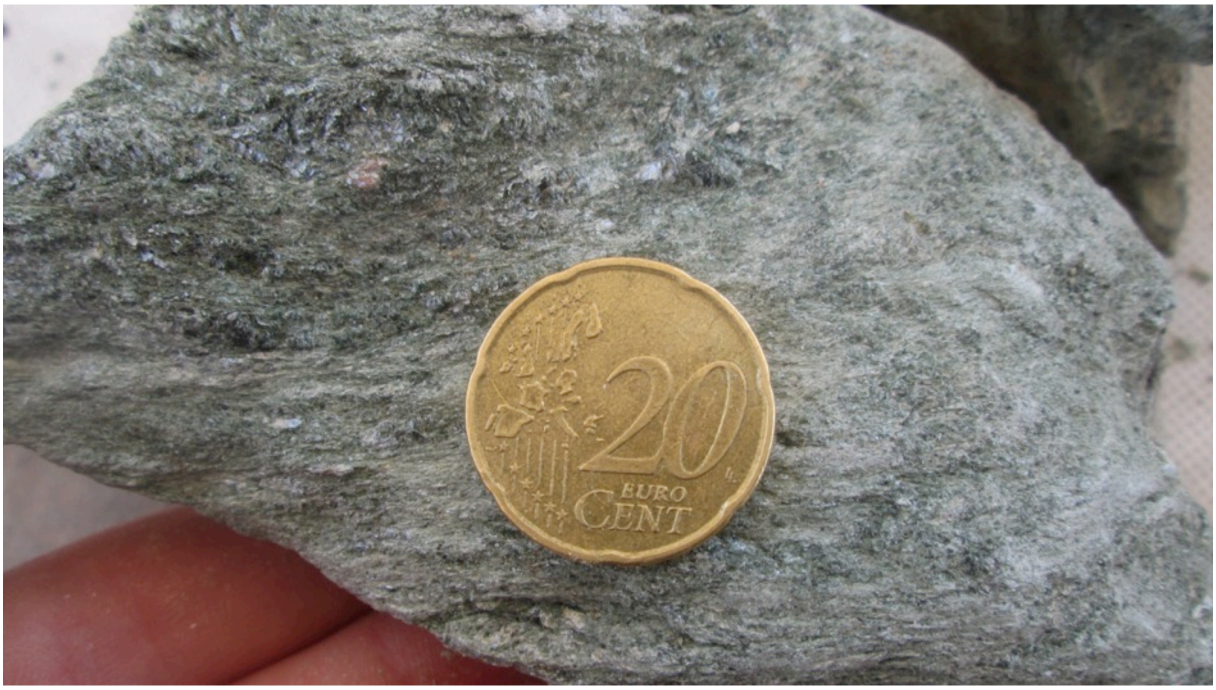

Figure S2-1. Sample 14HSY-7B. Strongly deformed, fine-grained chlorite schist.

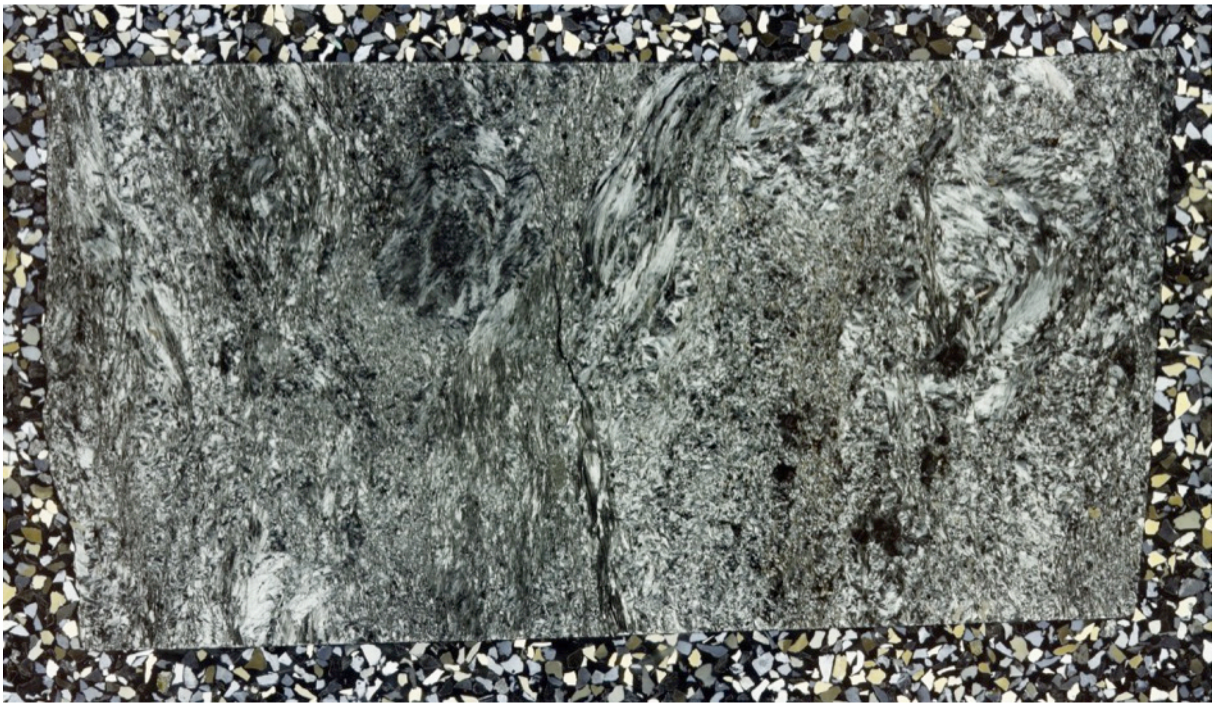

Figure S2-2. Thin section photograph of sample 14HSY-7B using crossed polarizers (length of the thin section ~4.5 cm). Strongly deformed chlorite schist containing thin layers of carbonate and abundant titanite (see Fig. 3-1 for hand sample image).

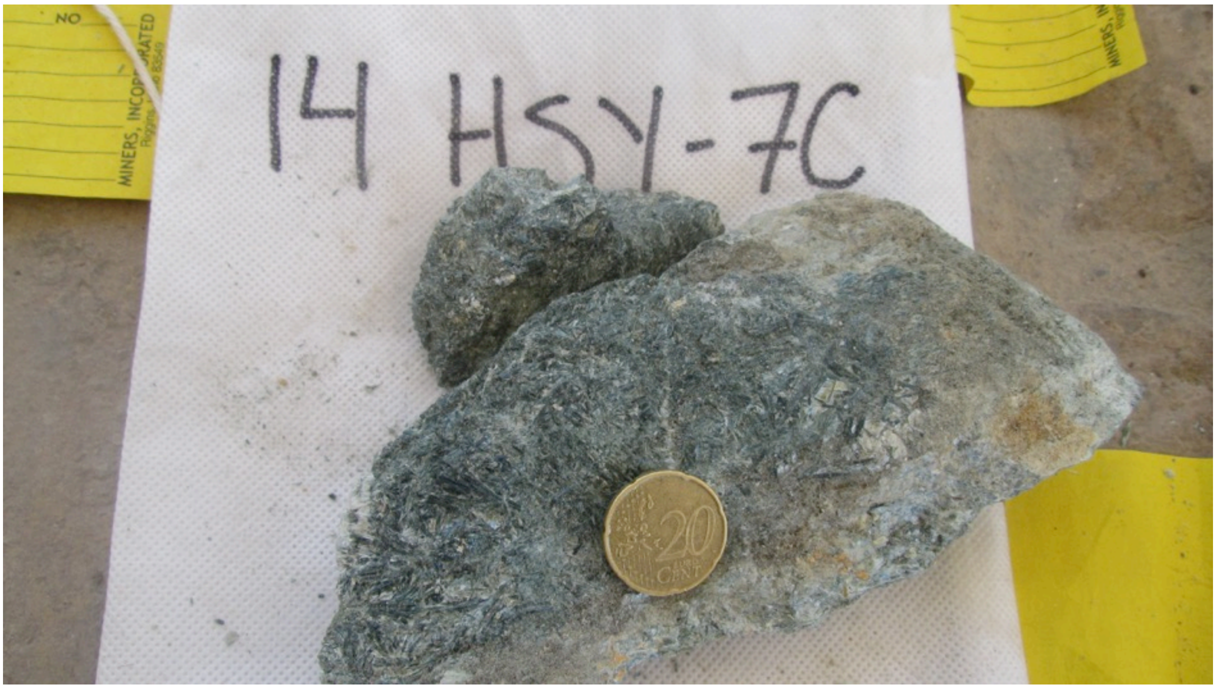

Figure S2-3. Sample 14HSY-7C. Chlorite-amphibole schist.

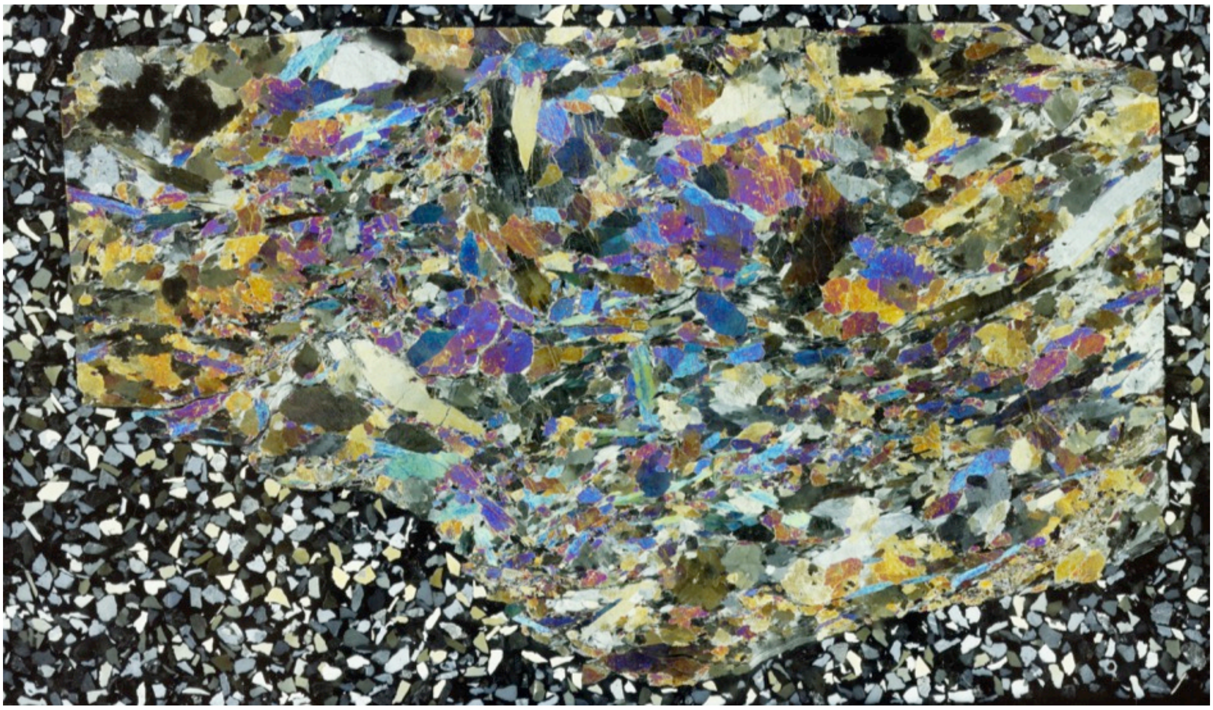

Figure S2-4. Thin section photograph of sample 14HSY-7C using crossed polarizers (length of the thin section ~4.5 cm). Chlorite-amphibole schist with euhedral amphibole (tremolite) overgrowing chlorite (see Fig. 3-3 for hand sample image).

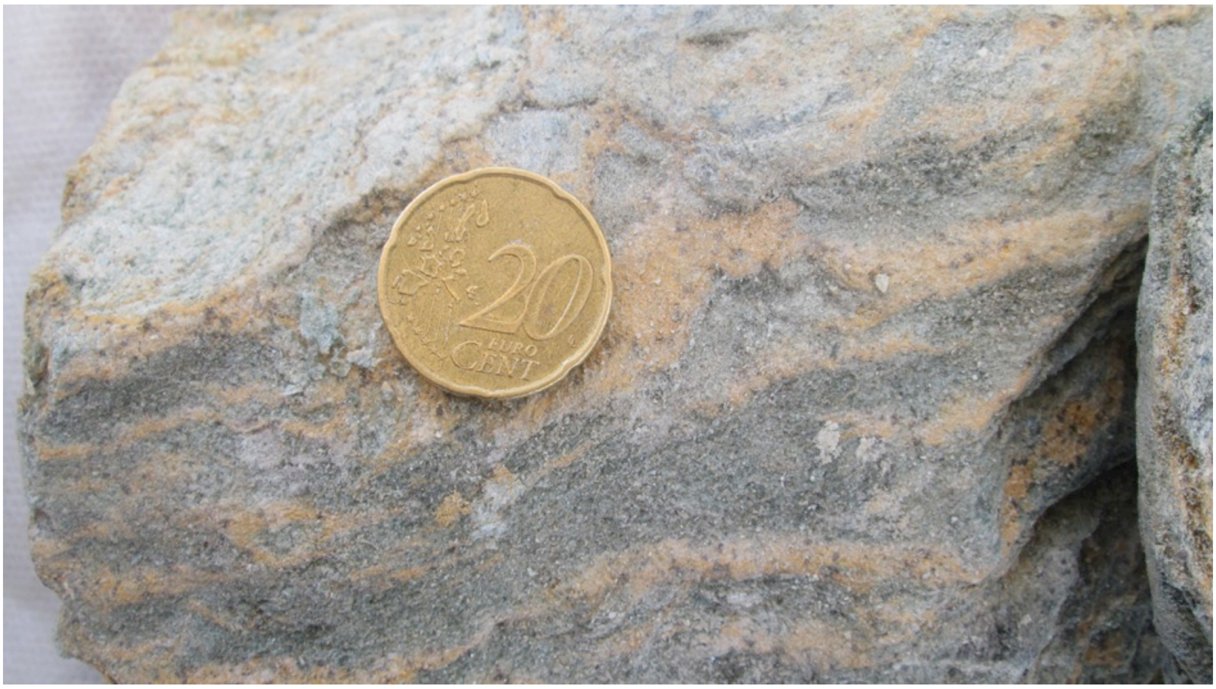

Figure S2-5. 14HSY-7E. Chlorite-amphibole schist with dolomite layers.

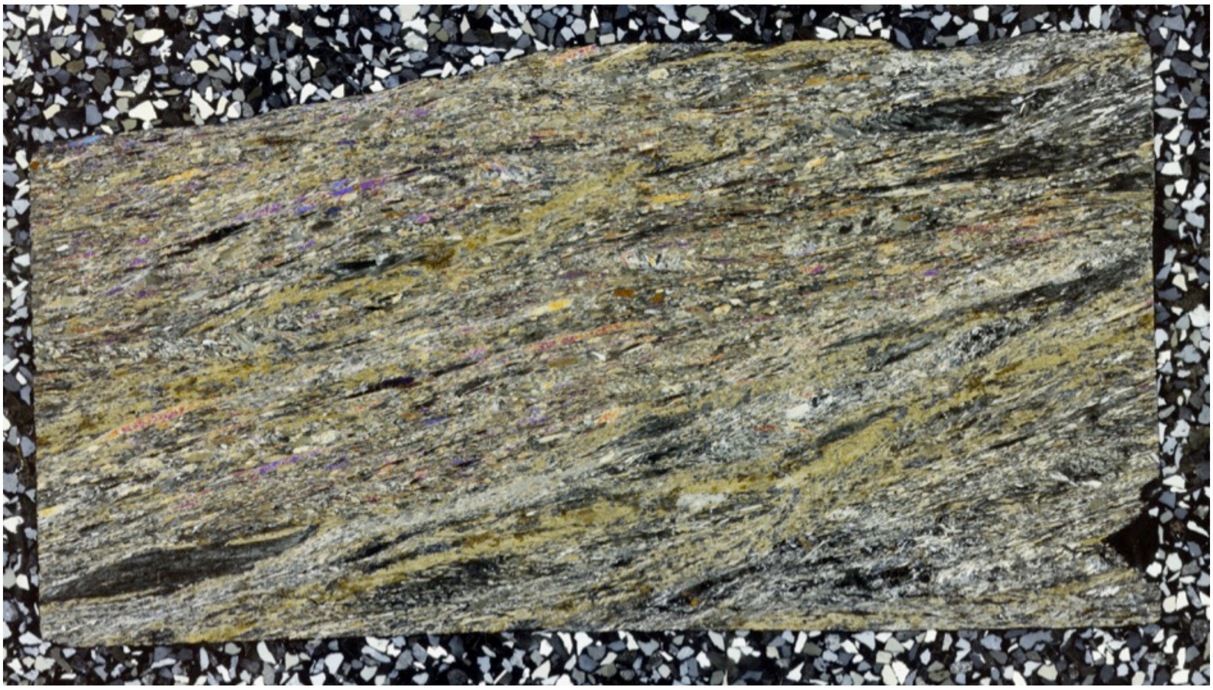

Figure S2-6. Thin section photograph of sample 14HSY-7E using crossed polarizers (length of the thin section ~4.5 cm). Serpentine-bearing chlorite-amphibole schist with dolomite-layers (see Fig. 3-5 for hand sample image).

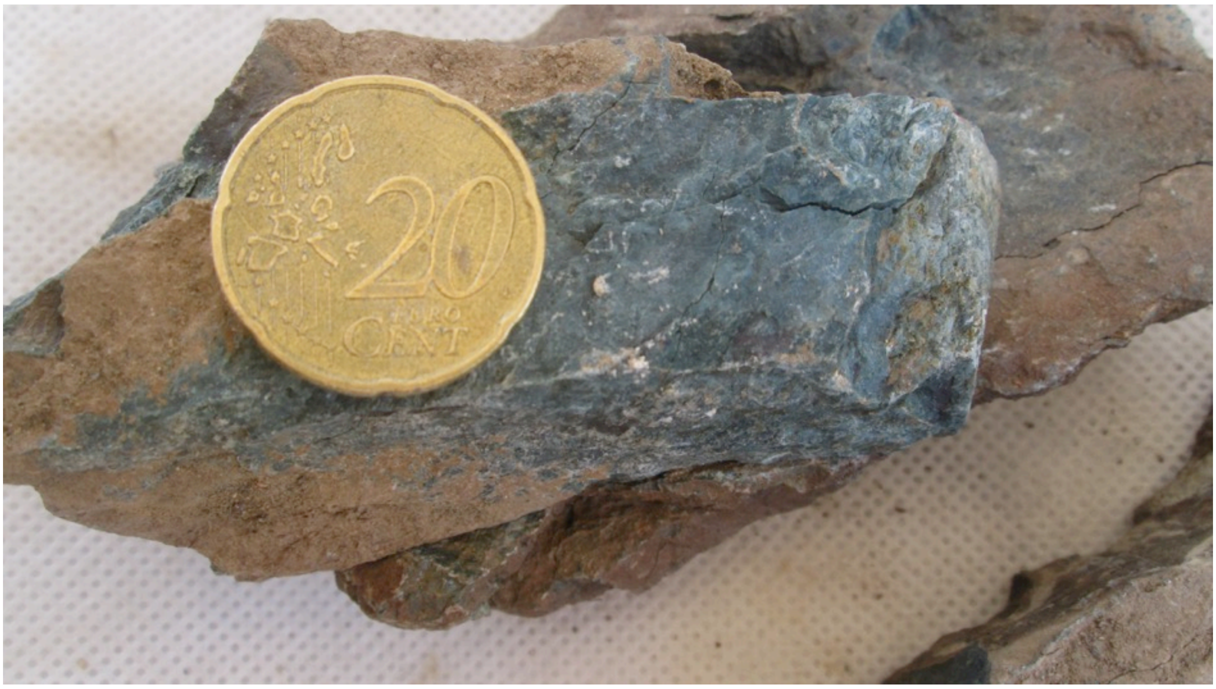

Figure S2-7. Sample 14BSY-22B. Massive, fine-grained serpentinite.

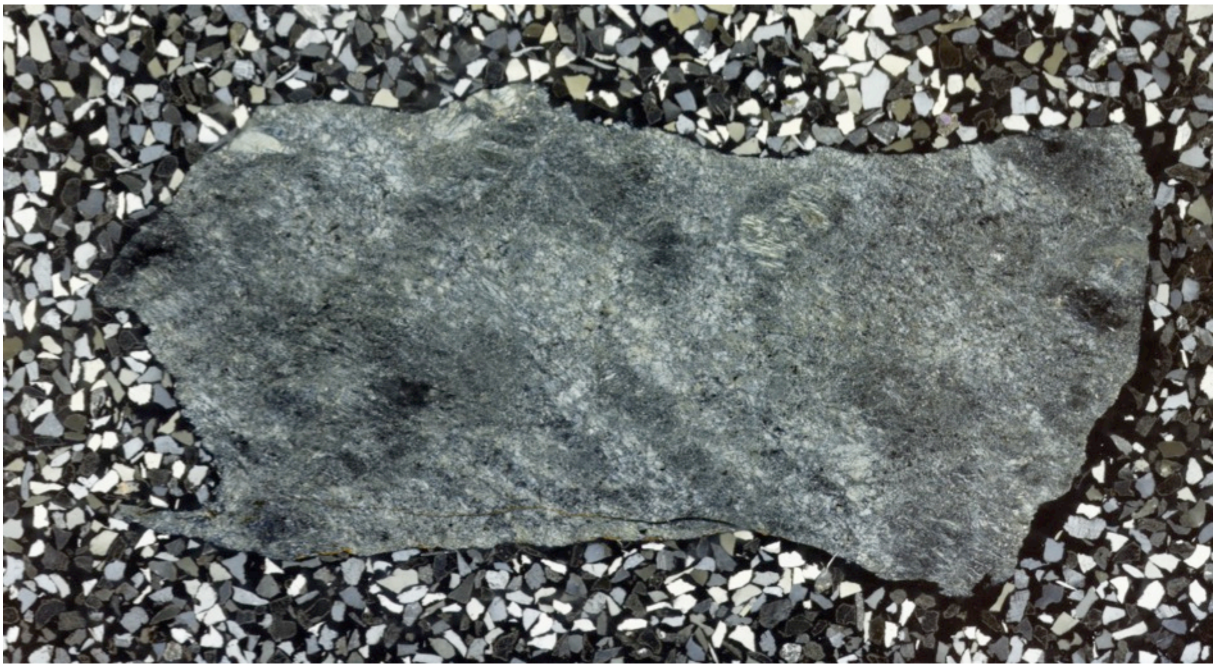

Figure S2-8. Thin section photograph of sample 14BSY-22B using crossed polarizers (length of the thin section ~4.5 cm). Fine-grained serpentinite with local bastites preserved and rare fine-grained amphibole in the groundmass (see Fig. 3-7 for hand sample image).

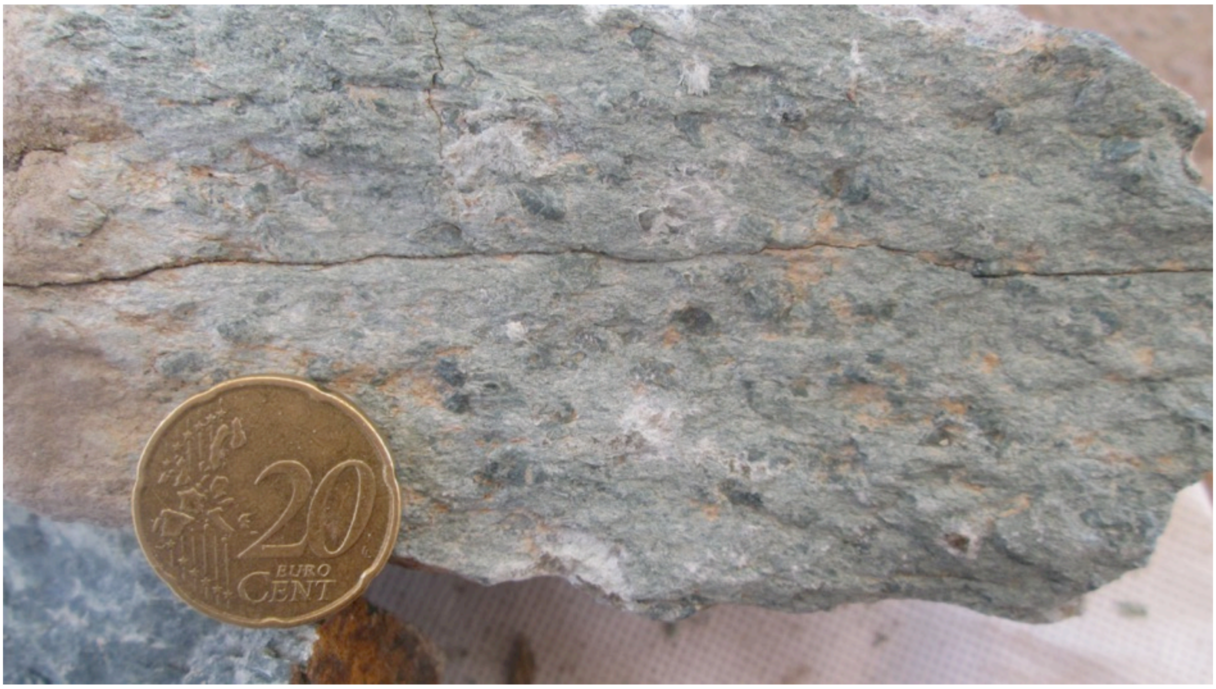

Figure S2-9. Sample 14CSY-30A. Fine-grained, talc-bearing serpentinite, locally with bastites (pseudomorphs of pyroxene) preserved.

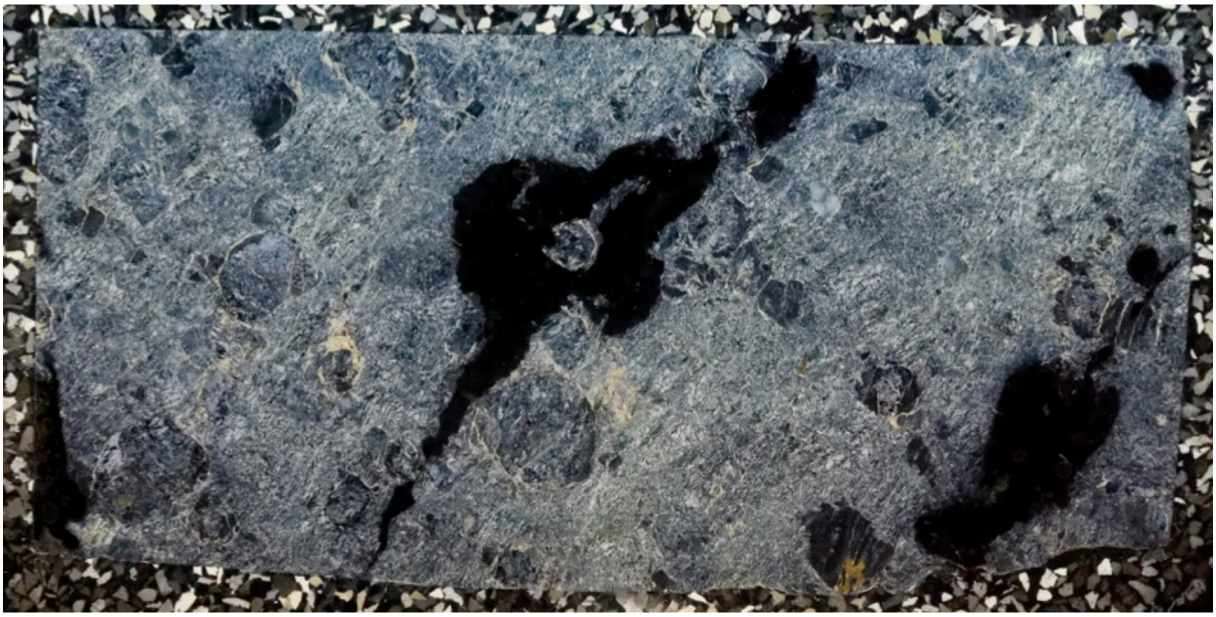

Figure S2-10. Thin section photograph of sample 14CSY-30A using crossed polarizers (length of the thin section ~4.5 cm). Fine-grained serpentinite with bastites preserved. Serpentine was confirmed as antigorite using Raman spectroscopy. Traces of talc are finely intergrown with serpentine or as thin veins (see Fig. 3-9 for hand sample image).

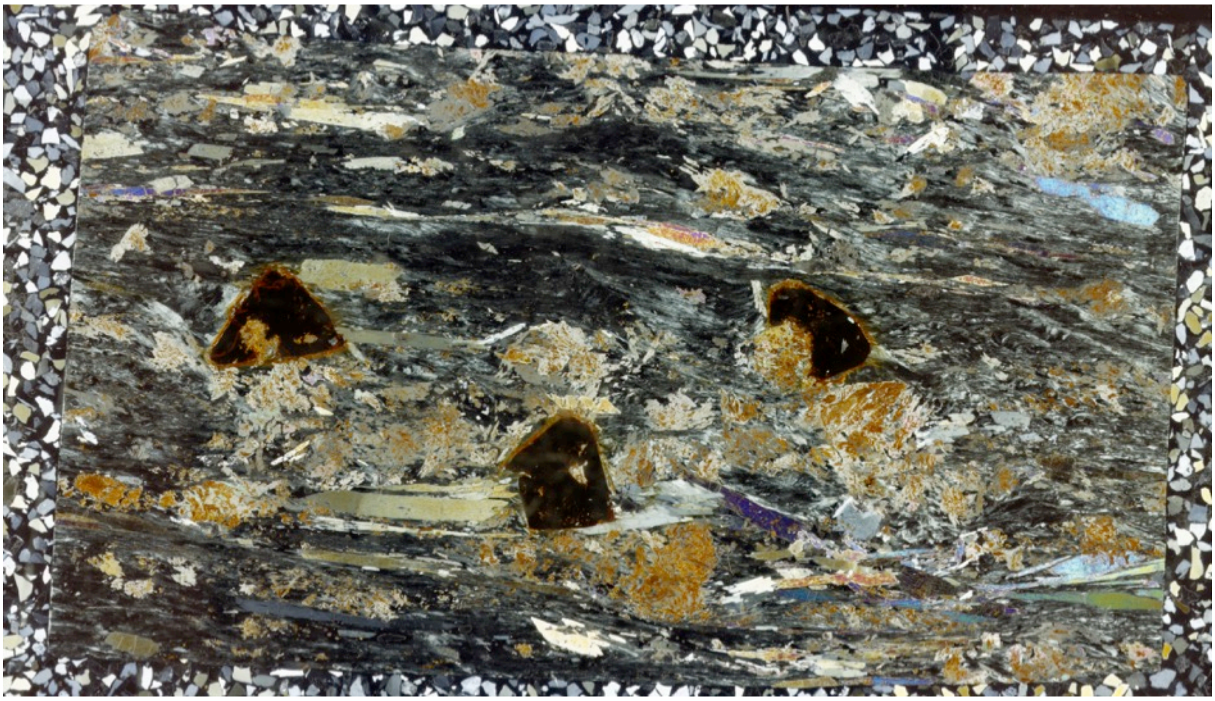

Figure S2-11. Thin section photograph of sample 14HSY-43B using crossed polarizers (length of the thin section ~4.5 cm). Serpentinite overgrown by zoned glaucophane needles and euhedral hematite crystals.

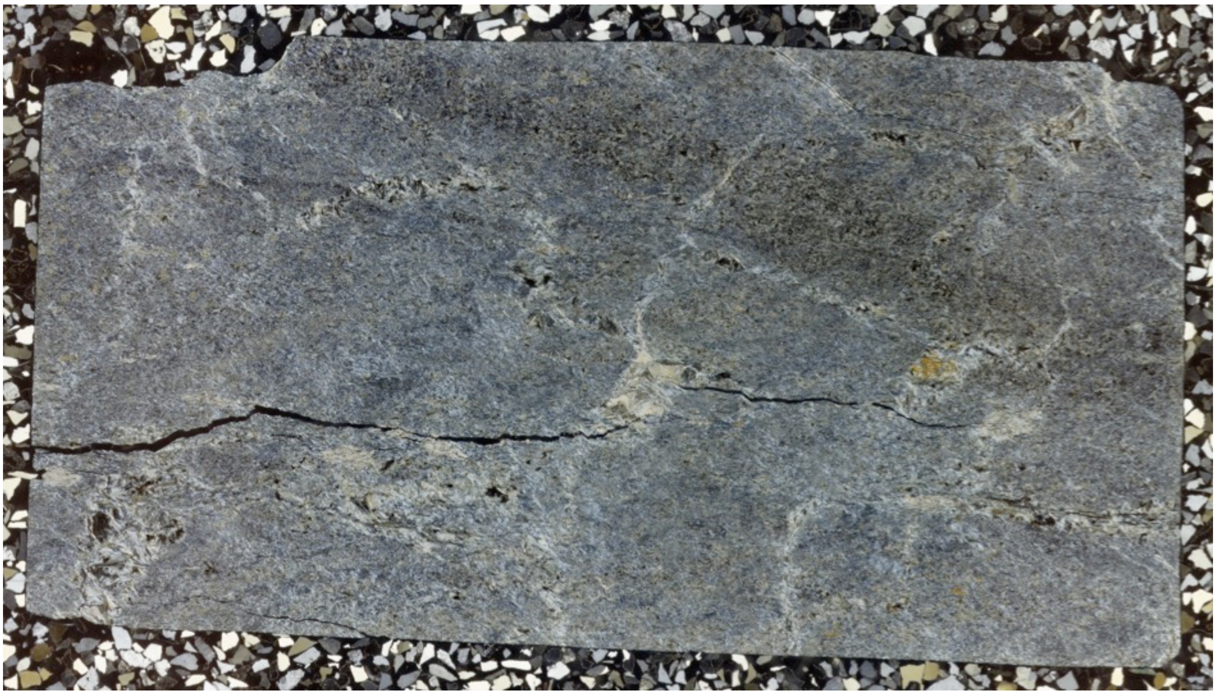

Figure S2-12. Thin section photograph of sample 14RSY-56A using crossed polarizers (length of the thin section ~4.5 cm). Talc-bearing, fine-grained serpentinite with carbonate as pockets. Note, this sample has the highest Li, Rb, and Cs concentrations, $\delta^{13}C_{TIC}$ values of -2.2‰ and $\delta^{34}S_{sulphate}$ values of 15.6‰ and thus suggests fluid ingress from dehydrating pelagic sediments. The presence of thin talc and carbonate veins further supports fluid input during exhumation.

3 Microprobe analyses of carbonate-bearing samples

Selected EMP analyses of carbonate minerals are shown in Tables S2 and S3. Figures 3-1 to 3-6 show backscattered electron (BSE) images of selected carbonate-bearing samples showing the occurrence of the carbonates and the mineral parageneses. Abbreviations are: Cc = calcite, Dol = dolomite, Chl = chlorite, Trem = tremolite, Tlc = talc, Qz = quarz, Serp = serpentine, Mgt = magnetite.

Table S2. Selected EMP analyses of calcite mineral phases.

(in wt.%)	14BSY-33A_P7	14BSY-33A_P8	14BSY-33A_P14	14BSY-33A_P15	14BSY-33A_P18	14BSY-33A_P19	14BSY-33A_P21	14BSY-33A_P23	14BSY-33A_P24	14BSY-33A_P27	14BSY-33A_P28	14HSY-7C_P1	14HSY-7C_P2	14HSY-7C_P10
SiO_2	0.00	0.00	0.15	0.00	0.00	0.00	0.00	0.05	0.05	0.06	0.00	0.00	0.00	0.00
FeO	0.00	0.00	0.22	0.15	0.00	0.09	1.06	0.17	0.19	0.13	0.14	0.13	0.09	0.12
CaO	55.56	56.66	58.00	56.40	56.05	55.15	54.82	56.13	55.08	55.48	56.10	56.14	56.85	55.63
Na_2O	0.00	0.00	0.00	0.00	0.00	0.00	0.00	0.00	0.00	0.00	0.00	0.00	0.00	0.00
Cr_2O_3	0.00	0.00	0.00	0.00	0.00	0.00	0.00	0.00	0.00	0.00	0.00	0.00	0.00	0.00
Al_2O_3	0.00	0.00	0.00	0.00	0.00	0.00	0.00	0.00	0.00	0.00	0.00	0.00	0.00	0.00
K_2O	0.00	0.00	0.00	0.00	0.00	0.00	0.00	0.00	0.00	0.00	0.00	0.00	0.00	0.00
MgO	0.00	0.00	0.22	0.00	0.32	0.11	0.63	0.22	0.29	0.25	0.26	0.36	1.02	1.12
TiO_2	0.00	0.00	0.00	0.00	0.00	0.00	0.00	0.00	0.00	0.00	0.00	0.00	0.00	0.00
Total	55.56	56.66	58.59	56.54	56.37	55.35	56.50	56.58	55.61	55.90	56.50	56.63	57.96	56.88

Table S3. Selected EMP analyses of dolomite mineral phases.

(in wt.%)	14HSY-7E_P6	14HSY-7E_P7	14BSY-33A_P2	14BSY-33A_P4	14BSY-33A_P22	14HSY-7C_P4	14HSY-7C_P5	14HSY-7C_P12	14RSY-56A_P4	14RSY-56A_P5	14RSY-56A_P9
SiO_2	<l.o.d.	0.12	<l.o.d.	<l.o.d.	<l.o.d.	<l.o.d.	<l.o.d.	<l.o.d.	<l.o.d.	<l.o.d.	<l.o.d.
FeO	3.20	2.60	4.51	4.45	4.65	0.40	0.57	0.69	2.02	2.06	2.31
CaO	29.93	29.55	28.91	29.24	28.95	32.37	32.61	32.24	30.02	29.95	30.32
Na_2O	<l.o.d.	<l.o.d.	<l.o.d.	<l.o.d.	<l.o.d.	<l.o.d.	<l.o.d.	<l.o.d.	<l.o.d.	<l.o.d.	<l.o.d.
Cr_2O_3	<l.o.d.	<l.o.d.	<l.o.d.	<l.o.d.	<l.o.d.	<l.o.d.	<l.o.d.	<l.o.d.	<l.o.d.	<l.o.d.	<l.o.d.
Al_2O_3	<l.o.d.	0.05	<l.o.d.	<l.o.d.	<l.o.d.	<l.o.d.	<l.o.d.	<l.o.d.	<l.o.d.	<l.o.d.	<l.o.d.
K_2O	<l.o.d.	<l.o.d.	<l.o.d.	<l.o.d.	<l.o.d.	<l.o.d.	<l.o.d.	<l.o.d.	<l.o.d.	<l.o.d.	<l.o.d.
MgO	20.38	19.97	19.30	18.94	18.68	21.19	20.98	18.62	21.31	21.32	21.56
TiO_2	<l.o.d.	<l.o.d.	<l.o.d.	<l.o.d.	<l.o.d.	<l.o.d.	<l.o.d.	<l.o.d.	<l.o.d.	<l.o.d.	0.23
Total	53.50	52.30	52.72	52.63	52.28	53.95	54.16	51.56	53.35	53.33	54.41

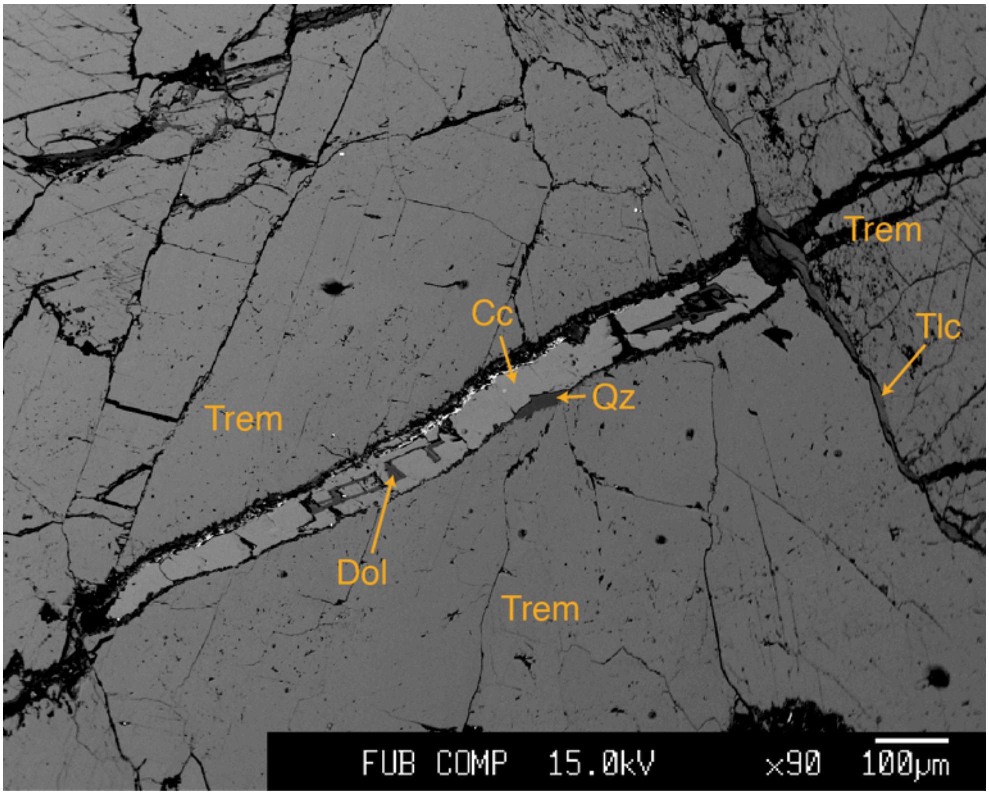

Figure S3-1. Sample 14HSY-7C (chlorite-amphibole schist). BSE image of a carbonate vein in tremolite. The carbonate vein mostly consists of calcite with rare dolomite along some thin fractures.

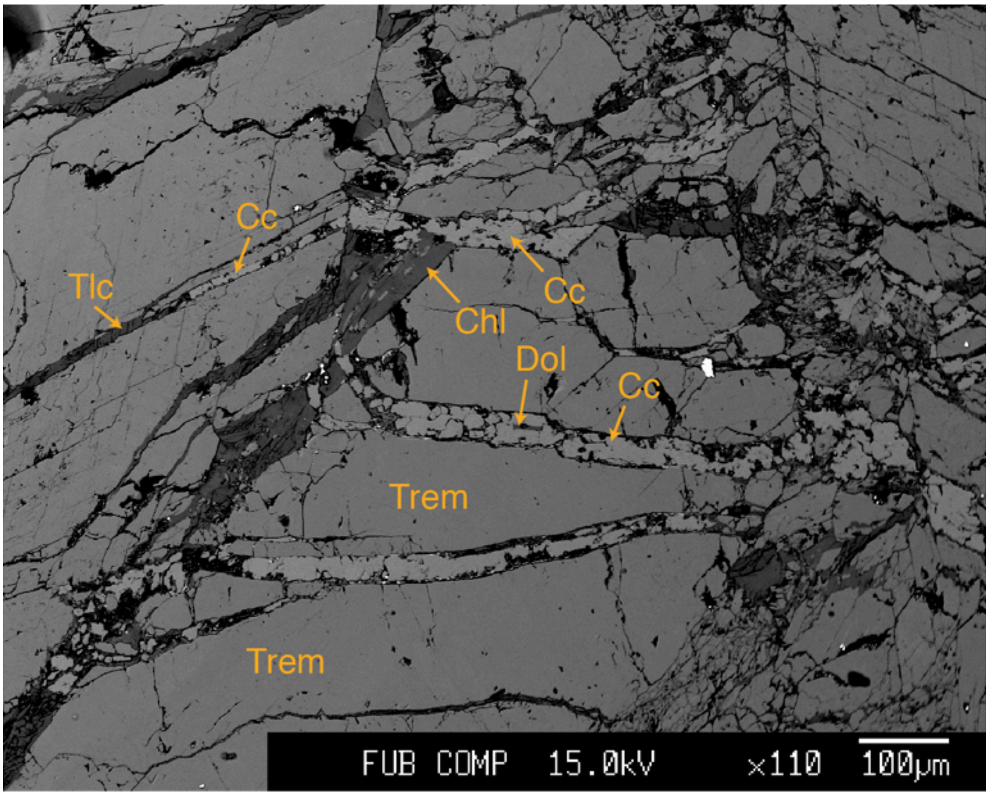

Figure S3-2. Sample 14HSY-7C (chlorite-amphibole schist). BSE image of carbonate veins in tremolite. The carbonate veins mostly consist of calcite and rare dolomite. Locally rare talc veins and chlorite occur in tremolite.

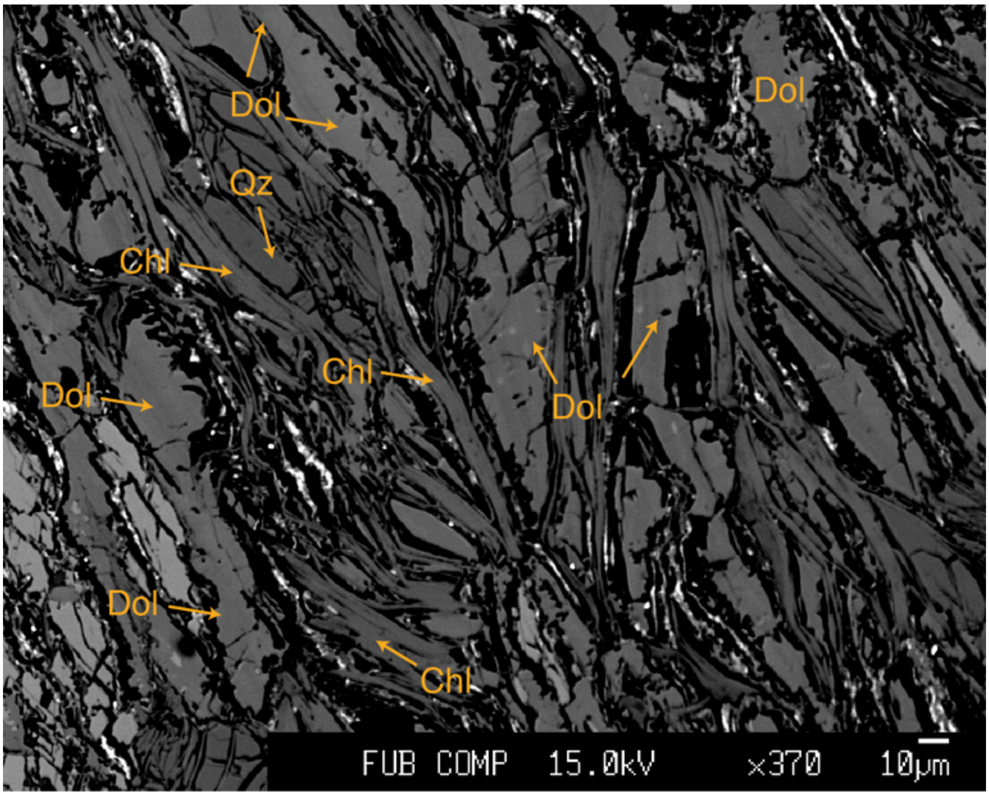

Figure S3-3. Sample 14HSY-7E (chlorite-amphibole schist). BSE image of dolomite intergrown within fibrous chlorite and quartz.

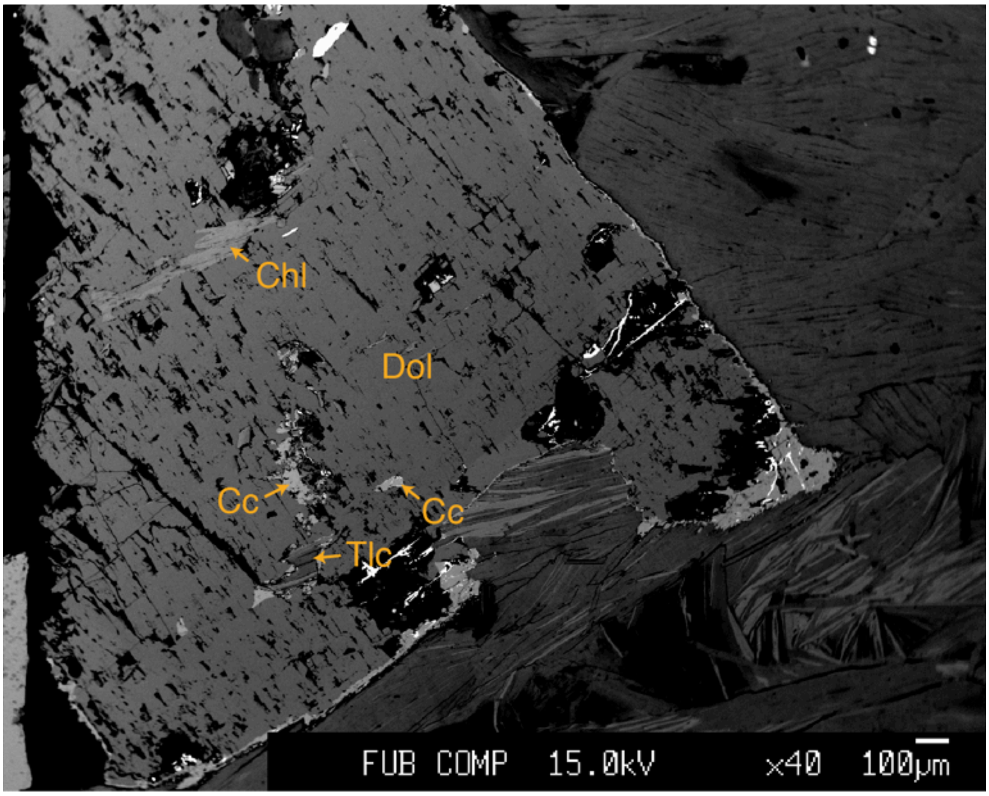

Figure S3-4. Sample 14BSY-33A (dolomite-bearing mica schist). BSE image of euhedral dolomite in a mica schist showing inclusions of chlorite, calcite, and talc within the euhedral dolomite.

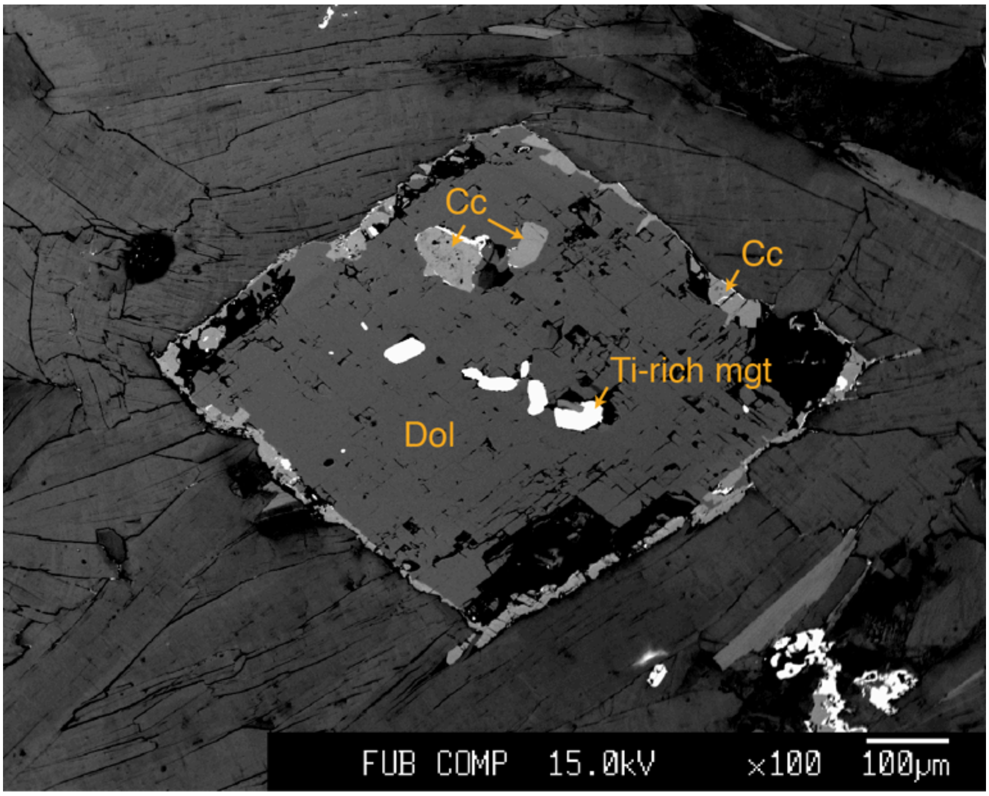

Figure S3-5. Sample 14BSY-33A (dolomite-bearing mica schist). BSE image of euhedral carbonate in a mica schist showing calcite as inclusions and along the rim of the dolomite crystal.

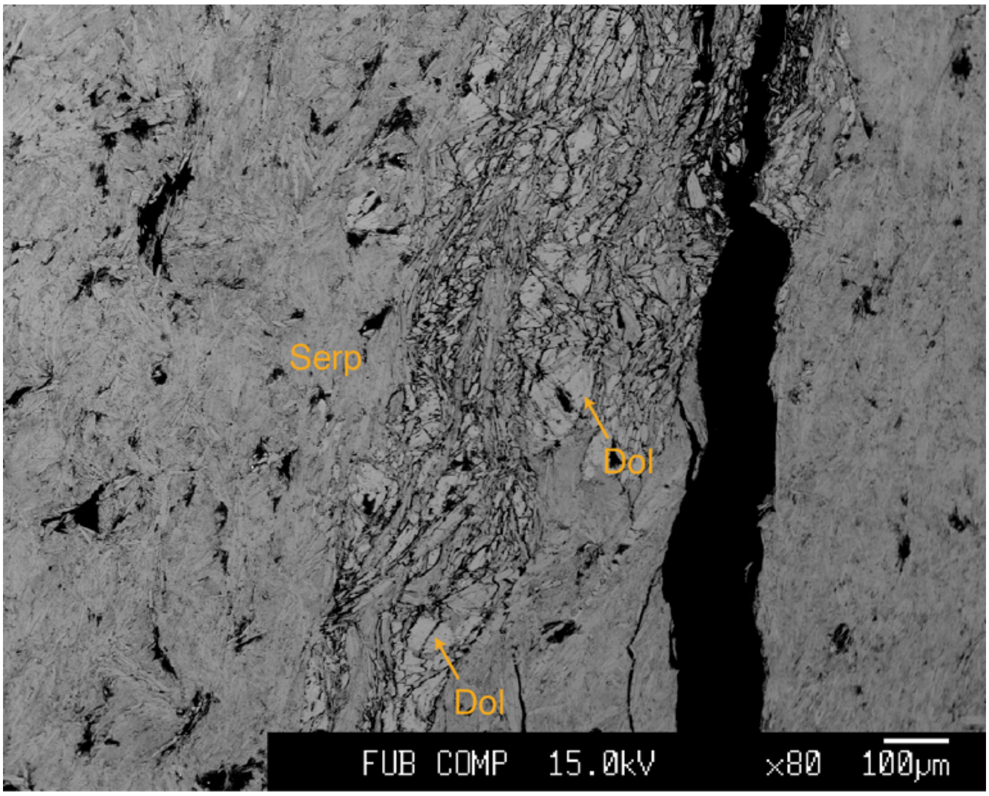

Figure S3-6. Sample 14RSY-56A (serpentinite). BSE image of talc-bearing, fine-grained serpentinite with dolomite as pockets in the groundmass.

4 Major and trace element compositions

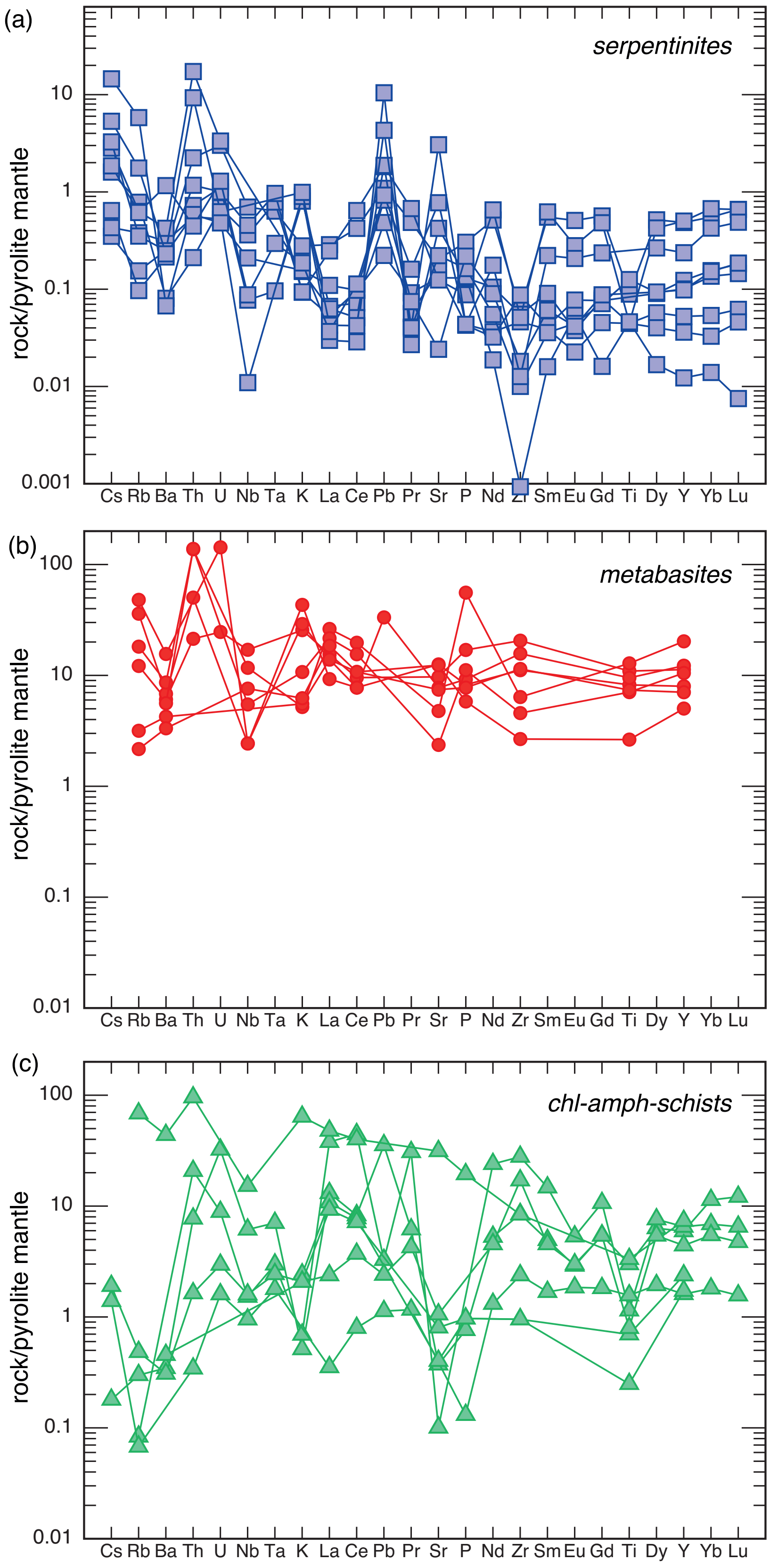

Figure S4-1. Trace element patterns of (a) the serpentinites, (b) the metabasic samples, and (c) the chlorite amphibole schists normalized to pyrolite mantle (McDonough and Sun, 1995).

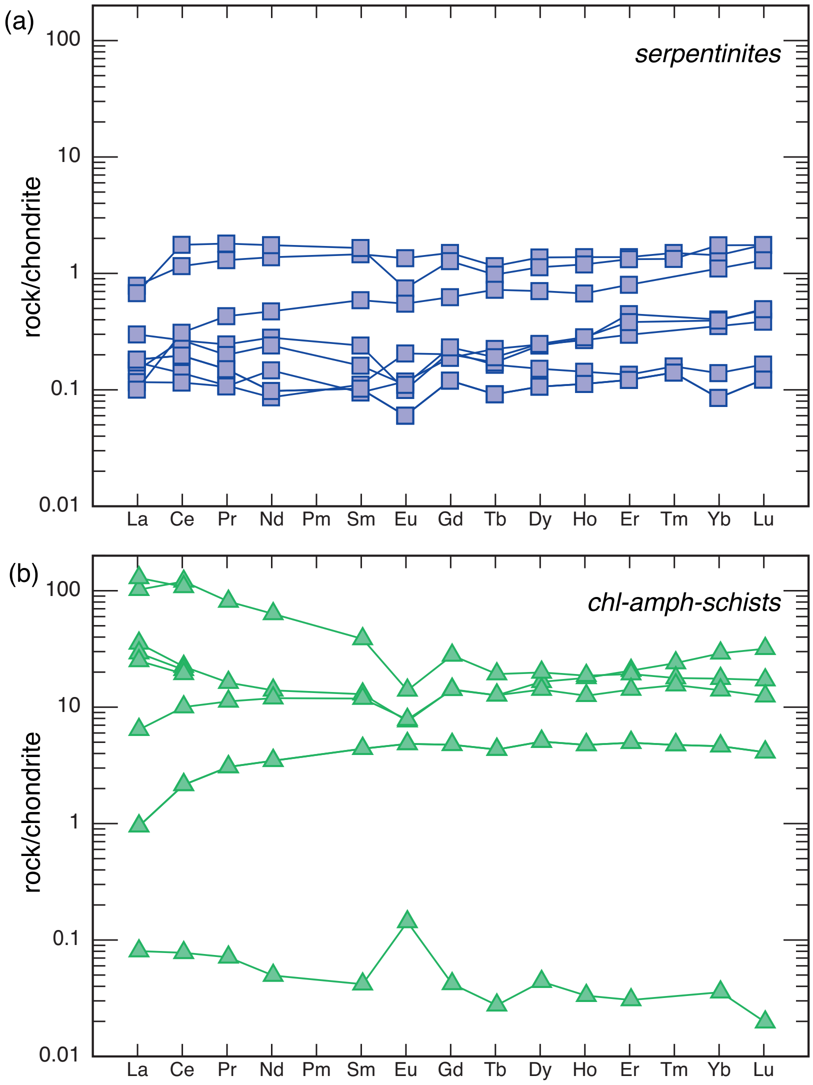

Figure S4-2. Rare earth element (REE) patterns of (a) the serpentinites, and (b) the chlorite amphibole schists normalized to chondrite (Sun and McDonough, 1989).

Table S4. Bulk rock major and trace element compositions

sample type	serpentinite	serpentinite	serpentinite	serpentinite	serpentinite	serpentinite w/ talc	serpentinite w/ talc	serpentinite w/ talc
Specimen	**14BSY-22B***	**14CSY-30A***	*14HSY-43B†*	**14RSY-56A***	**14KA01***	**13KA03***	**14FI02***	**14FI03***
(in wt.%)								
**SiO2**	42.12	41.91	33.14	42.14	42.76	52.53	42.18	32.36
**TiO2**	0.02	0.01	9.63	0.01	0.01	0.01	0.00	0.03
**Al2O3**	2.72	2.63	15.26	2.24	2.85	3.19	1.75	3.15
**Fe2O3T**	8.95	9.17	14.06	8.15	9.95	6.07	6.72	8.22
**MnO**	0.13	0.13	0.17	0.08	0.15	0.06	0.10	0.19
**MgO**	33.96	35.05	22.32	35.68	33.65	29.89	33.28	32.89
**CaO**	0.71	0.09	4.82	0.20	0.12	0.05	2.99	6.09
**Na2O**	0.08	0.04	0.32	0.05	0.08	0.26	0.12	0.26
**K2O**	0.01	0.00	0.00	0.02	0.00	0.03	0.00	0.03
**P2O5**	0.01	0.00	0.02	0.00	0.00	0.00	0.00	0.00
**Total**	**100.29**	**100.49**	**99.74**	**100.22**	**100.07**	**99.73**	**100.68**	**101.79**
**LOI**	11.59	11.46	9.56	11.65	10.50	7.64	13.52	18.59
**FeO**			10.25					
**Fe2O3**			2.67					
**FeO/FeT**			0.79					
(in ppm)								
**Rb**	0.09	0.47	<0.5	3.48	0.06	0.23	0.38	0.21
**Sr**	4.06	2.61	14.00	3.09	2.48	8.41	60.80	4.40
**Y**	2.09	0.23	13.90	0.16	2.16	0.05	0.53	0.43
**Zr**	0.65	0.58	58.00	0.48	0.54	0.01	0.91	0.11
**V**	31.91	56.06	144.00	36.00	51.65	54.68	35.20	31.43
**Ni**	1729.70	2133.32	396.00	2516.76	2224.92	2132.38	2092.36	1727.20
**Cr**	2059.49	2747.12	132.00	2842.30	2765.75	3273.47	2486.12	1791.98
**Nb**	0.46	0.24	17.20	0.05	0.30	0.01	b.d.l.	b.d.l.
**Ga**			14.50					
**Cu**	7.95	2.86	70.00	1.93	7.58	6.08	4.16	2.33
**Zn**	77.86	107.97	133.00	187.51	96.69	63.00	44.74	51.34
**Co**	62.33	70.27	116.00	84.80	79.90	98.18	84.68	79.01
**Ba**	1.65	0.53	<3	0.44	1.42	2.04	2.79	1.64
**La**	0.19	0.03	11.00	0.04	0.16	0.02	0.04	0.03
**Ce**	0.71	0.16	6.00	0.12	1.08	0.05	0.09	0.07
**U**	0.06	0.01	1.70	0.02	0.02	0.01	0.07	0.01
**Th**	0.18	0.02	21.90	0.06	0.09	0.04	1.37	0.74
**Sc**	14.05	11.99	13.00	10.64	6.42	7.65	8.80	12.61
**Pb**	0.16	0.03	<1	0.07	0.27	1.57	0.28	0.64
**As**	4.40	1.30	n.d.	2.56	2.34	0.46	16.14	1.10
**Mo**	b.d.l.	b.d.l.	n.d.	0.58	b.d.l.	0.35	0.18	0.28
**Cs**	0.01	0.03	n.d.	0.31	0.01	0.01	0.06	0.07
**Li**	2.17	12.72	n.d.	4.54	2.04	0.43	1.08	0.98
**Pr**	0.12	0.02	n.d.	0.01	0.17	0.01	0.01	0.01
**Nd**	0.64	0.13	n.d.	0.05	0.82	0.02	0.04	0.07
**Sm**	0.22	0.04	n.d.	0.02	0.25	0.01	0.02	0.01
**Eu**	0.08	0.01	n.d.	0.00	0.04	0.01	0.01	0.01
**Tb**	0.04	0.01	n.d.	0.00	0.04	0.00	0.01	0.01
**Gd**	0.31	0.04	n.d.	0.02	0.26	0.01	0.04	0.04
**Dy**	0.35	0.04	n.d.	0.03	0.29	0.01	0.06	0.06
**Ho**	0.08	0.01	n.d.	0.01	0.07	0.00	0.02	0.02
**Er**	0.23	0.02	n.d.	0.02	0.22	0.01	0.07	0.05
**Tm**	0.04	0.00	n.d.	0.00	0.03	b.d.l.	b.d.l.	b.d.l.
**Yb**	0.24	0.02	n.d.	0.01	0.30	0.01	0.07	0.06
**Lu**	0.04	0.00	n.d.	0.00	0.04	0.00	0.01	0.01
**Hf**	0.04	0.03	n.d.	0.01	0.04	0.00	0.02	0.01
**Ta**	0.02	0.03	n.d.	0.00	0.04	b.d.l.	b.d.l.	b.d.l.
**W**	b.d.l.	b.d.l.	n.d.	0.09	b.d.l.	0.05	0.15	0.08
**Th/U**	2.91	1.20	n.d.	2.56	4.58	4.13	20.30	58.23
**La_N/Sm_N**	0.52	0.59	n.d.	1.72	0.40	1.86	1.50	1.19
**La_N/Yb_N**	0.52	0.99	n.d.	2.02	0.37	2.14	0.40	0.31
**Sm_N/Yb_N**	1.00	1.69	n.d.	1.18	0.93	1.15	0.27	0.26

N = normalized to C1 (McDonough and Sun, 1995)
*** mark samples measured by Cooperdock et al., 2018**
† mark samples measured in this study

Table S4. *continued.*

**sample type**	serpentinite w/ talc	serpentinite	blueschist	pillow basalt	basaltic dike	metagabbro	blueschist	metagabbro
Specimen	**14KIN01***	**13KIN01***	*14BSY-07D†*	*14BSY-31A†*	*14BSY-34A†*	*14HSY-42A†*	*14GSY-47A†*	*14KSY-59A†*
(in wt.%)								
**SiO2**	42.34	42.11	49.48	52.03	46.66	51.05	48.03	54.05
**TiO2**	0.00	0.00	2.20	1.65	2.58	0.53	1.91	1.42
**Al2O3**	2.57	2.47	15.32	16.66	14.08	14.54	19.21	15.76
**Fe2O3T**	9.68	9.25	11.60	9.57	18.37	7.96	9.78	9.84
**MnO**	0.16	0.14	0.22	0.18	0.83	0.15	0.27	0.20
**MgO**	34.28	34.73	5.65	7.31	2.11	9.23	4.26	3.03
**CaO**	0.04	0.04	9.88	6.27	6.36	12.55	12.28	3.82
**Na2O**	0.08	0.09	4.73	5.05	7.82	3.89	3.36	11.44
**K2O**	0.00	0.01	0.74	1.25	0.15	0.16	0.84	0.31
**P2O5**	0.00	0.00	0.35	0.17	1.15	0.12	0.19	0.23
**Total**	**99.91**	**100.09**	**100.17**	**100.14**	**100.11**	**100.18**	**100.13**	**100.10**
**LOI**	10.77	11.25	2.03	2.60	1.10	2.64	4.20	0.62
**FeO**			6.53	4.68	9.24	3.24	3.26	2.70
**Fe2O3**			4.34	4.37	8.10	4.36	6.16	6.84
**FeO/FeT**			0.60	0.52	0.53	0.43	0.35	0.28
(in ppm)								
**Rb**	1.06	0.37	7.30	21.60	10.90	1.90	28.80	<0.5
**Sr**	0.48	15.41	193.00	250.00	95.00	246.00	154.00	47.00
**Y**	0.42	1.02	48.50	34.30	87.20	21.60	52.60	45.20
**Zr**	0.19	0.13	216.00	117.00	67.00	28.00	165.00	48.00
**V**	57.99	50.84	263.00	211.00	150.00	218.00	288.00	37.00
**Ni**	1936.01	2196.00	68.00	108.00	65.00	75.00	132.00	24.00
**Cr**	2113.52	2845.35	159.00	239.00	73.00	122.00	269.00	54.00
**Nb**	0.14	0.06	11.20	1.60	7.70	<0.5	1.60	3.60
**Ga**			18.30	14.60	18.20	12.90	22.70	30.00
**Cu**	1.38	5.04	48.00	73.00	95.00	276.00	66.00	23.00
**Zn**	76.95	81.73	93.00	106.00	163.00	142.00	85.00	55.00
**Co**	112.33	117.47	38.00	41.00	37.00	40.00	26.00	17.00
**Ba**	1.52	7.66	40.00	45.00	57.00	28.00	103.00	37.00
**La**	0.07	0.02	10.00	6.00	17.00	9.00	9.00	14.00
**Ce**	0.16	0.19	16.00	13.00	33.00	18.00	13.00	26.00
**U**	0.01	0.03	0.50	<0.5	<0.5	<0.5	2.90	<0.5
**Th**	0.05	0.04	1.70	4.00	10.90	<0.5	<0.5	11.00
**Sc**	12.94	8.79	30.00	23.00	23.00	49.00	36.00	14.00
**Pb**	0.12	0.14	<1	<1	<1	<1	5.00	<1
**As**	1.55	0.99	n.d.	n.d.	n.d.	n.d.	n.d.	n.d.
**Mo**	0.06	0.05	n.d.	n.d.	n.d.	n.d.	n.d.	n.d.
**Cs**	0.11	0.04	n.d.	n.d.	n.d.	n.d.	n.d.	n.d.
**Li**	1.45	1.16	n.d.	n.d.	n.d.	n.d.	n.d.	n.d.
**Pr**	0.02	0.04	n.d.	n.d.	n.d.	n.d.	n.d.	n.d.
**Nd**	0.11	0.22	n.d.	n.d.	n.d.	n.d.	n.d.	n.d.
**Sm**	0.02	0.09	n.d.	n.d.	n.d.	n.d.	n.d.	n.d.
**Eu**	0.01	0.03	n.d.	n.d.	n.d.	n.d.	n.d.	n.d.
**Tb**	0.01	0.03	n.d.	n.d.	n.d.	n.d.	n.d.	n.d.
**Gd**	0.05	0.13	n.d.	n.d.	n.d.	n.d.	n.d.	n.d.
**Dy**	0.06	0.18	n.d.	n.d.	n.d.	n.d.	n.d.	n.d.
**Ho**	0.02	0.04	n.d.	n.d.	n.d.	n.d.	n.d.	n.d.
**Er**	0.06	0.13	n.d.	n.d.	n.d.	n.d.	n.d.	n.d.
**Tm**	b.d.l.	b.d.l.	n.d.	n.d.	n.d.	n.d.	n.d.	n.d.
**Yb**	0.07	0.19	n.d.	n.d.	n.d.	n.d.	n.d.	n.d.
**Lu**	0.01	0.03	n.d.	n.d.	n.d.	n.d.	n.d.	n.d.
**Hf**	0.01	0.02	n.d.	n.d.	n.d.	n.d.	n.d.	n.d.
**Ta**	b.d.l.	0.01	n.d.	n.d.	n.d.	n.d.	n.d.	n.d.
**W**	0.05	0.01	n.d.	n.d.	n.d.	n.d.	n.d.	n.d.
**Th/U**	4.77	1.35	n.d.	n.d.	n.d.	n.d.	n.d.	n.d.
La_N/Sm_N	1.79	0.16	n.d.	n.d.	n.d.	n.d.	n.d.	n.d.
La_N/Yb_N	0.72	0.09	n.d.	n.d.	n.d.	n.d.	n.d.	n.d.
Sm_N/Yb_N	0.40	0.52	n.d.	n.d.	n.d.	n.d.	n.d.	n.d.

Table S4. *continued.*

**sample type**	blueschist	serpentine-talc-chlorite-schist	tlc-amph-chlorite-schist	Chlorite schist	Chlorite-amph-schist	Chlorite-amph-schist	Amph-schist	talc-rich serpentinite / chlorite-amph-schist
Specimen	*14BSY-68A†*	**14CSY-3D***	14CSY-3E*	**14HSY-7B***	*14HSY-7C†*	*14HSY-7E†*	*14CSY-12F†*	14HSY-19A*
(in wt.%)								
**SiO2**	48.45	44.81	*39.60*	28.94	53.40	50.93	50.18	*43.50*
**TiO2**	1.48	0.60	*0.23*	0.31	0.05	0.14	0.67	*0.16*
**Al2O3**	17.63	4.98	*8.30*	19.72	4.42	6.63	15.33	*4.80*
**Fe2O3T**	9.81	10.19	*2.40*	12.80	7.66	7.69	10.16	*6.00*
**MnO**	0.29	0.14	*0.12*	0.33	0.20	0.18	0.06	*0.13*
**MgO**	6.76	29.36	*22.10*	26.68	22.85	24.59	7.33	*23.30*
**CaO**	10.05	1.53	*3.80*	0.22	9.95	8.88	9.06	*5.00*
**Na2O**	4.88	0.12		0.08	1.25	0.87	5.00	*0.70*
**K2O**	0.18	0.01	*0.02*	0.00	0.07	0.06	1.85	*0.06*
**P2O5**	0.16	0.02		0.00	0.02	0.02	0.40	
**Total**	**99.69**	**99.80**		**100.11**	**99.87**	**99.99**	**100.04**	
**LOI**	7.27	8.03		11.02	4.47	8.49	7.24	
**FeO**	5.30				5.00	4.92	5.22	
**Fe2O3**	3.92				2.10	2.22	4.36	
**FeO/FeT**	0.57				0.70	0.69	0.54	
(in ppm)								
**Rb**	1.30	0.18	0.05	0.04	<0.5	<0.5	41.10	0.29
**Sr**	148.00	7.41	2.00	b.d.l.	8.00	16.00	623.00	21.00
**Y**	30.30	25.51	28.00	6.91	7.40	10.20	31.60	19.00
**Zr**	120.00	88.97	292.38	24.94	10.00	10.00	88.00	179.01
**V**	237.00	47.45	43.00	50.60	77.00	82.00	255.00	50.00
**Ni**	67.00	1857.80	1833.00	1740.18	1430.00	1516.00	65.00	2384.00
**Cr**	215.00	2837.43	2462.00	2136.72	1650.00	1677.00	114.00	3363.00
**Nb**	5.00	4.04	1.00	0.62	<0.5	<0.5	10.00	1.05
**Ga**	13.50				7.10	8.70	14.90	
**Cu**	270.00	3.46	4.20	1.03	34.00	49.00	103.00	3.10
**Zn**	664.00	91.81	83.00	197.02	63.00	59.00	82.00	71.00
**Co**	36.00	72.53	61.00	73.61	49.00	61.00	39.00	59.00
**Ba**	22.00	2.28	b.d.l.	b.d.l.	<3	3.00	289.00	2.03
**La**	12.00	8.49	24.55	0.23	7.00	6.00	31.00	1.54
**Ce**	18.00	13.85	74.62	1.34	13.00	12.00	67.00	6.22
**U**	<0.5	0.65	0.18	0.03	<0.5	<0.5	<0.5	0.06
**Th**	<0.5	0.61	1.65	0.03	<0.5	<0.5	7.60	0.13
**Sc**	27.00	12.55	9.09	3.07	10.00	9.00	25.00	13.69
**Pb**	<1	5.31	0.49	0.17	<1	<1	<1	0.36
**As**	n.d.	0.38	b.d.l.	0.02	n.d.	n.d.	n.d.	2.00
**Mo**	n.d.	b.d.l.	0.02	b.d.l.	n.d.	n.d.	n.d.	0.01
**Cs**	n.d.	0.00	0.00	0.03	n.d.	n.d.	n.d.	0.04
**Li**	n.d.	1.59	1.37	52.64	n.d.	n.d.	n.d.	2.75
**Pr**	n.d.	1.57	7.79	0.30	n.d.	n.d.	n.d.	1.08
**Nd**	n.d.	6.60	29.96	1.64	n.d.	n.d.	n.d.	5.66
**Sm**	n.d.	2.01	5.98	0.68	n.d.	n.d.	n.d.	1.84
**Eu**	n.d.	0.45	0.82	0.28	n.d.	n.d.	n.d.	0.46
**Tb**	n.d.	0.48	0.73	0.16	n.d.	n.d.	n.d.	0.48
**Gd**	n.d.	2.96	5.80	0.99	n.d.	n.d.	n.d.	2.94
**Dy**	n.d.	4.25	5.12	1.30	n.d.	n.d.	n.d.	3.65
**Ho**	n.d.	1.02	1.07	0.27	n.d.	n.d.	n.d.	0.72
**Er**	n.d.	3.46	3.23	0.83	n.d.	n.d.	n.d.	2.38
**Tm**	n.d.	0.62	0.46	0.12	n.d.	n.d.	n.d.	0.40
**Yb**	n.d.	5.01	3.03	0.80	n.d.	n.d.	n.d.	2.41
**Lu**	n.d.	0.82	0.44	0.11	n.d.	n.d.	n.d.	0.32
**Hf**	n.d.	2.28	5.72	0.68	n.d.	n.d.	n.d.	4.34
**Ta**	n.d.	0.26	0.11	0.07	n.d.	n.d.	n.d.	0.09
**W**	n.d.	0.15	0.70	b.d.l.	n.d.	n.d.	n.d.	0.04
**Th/U**	n.d.	0.94	9.09	0.84	n.d.	n.d.	n.d.	2.15
**La_N/Sm_N**	n.d.	2.64	2.56	0.21	n.d.	n.d.	n.d.	0.52
**La_N/Yb_N**	n.d.	1.15	5.51	0.19	n.d.	n.d.	n.d.	0.43
**Sm_N/Yb_N**	n.d.	0.44	2.15	0.93	n.d.	n.d.	n.d.	0.83

References

Cooperdock, E. H. G., Raia, N. H., Barnes, J. D., Stockli, D. F. & Schwarzenbach, E. M. Tectonic origin of serpentinites on Syros, Greece: Geochemical signatures of abyssal origin preserved in a HP/LT subduction complex. *Lithos*, 352-364 (2018).

McDonough, W.F., Sun, S.s. The composition of the Earth. *Chemical Geology* 120, 223-253 (1995).

Sun, S.S., McDonough, W.F. Chemical and isotopic systematics of oceanic basalts: implications for mantle composition and processes. *Geological Society London, Special Publications* 42, 313-345 (1989).
